# MUC1-C is a target of salinomycin in inducing ferroptosis of cancer stem cells

**DOI:** 10.1038/s41420-023-01772-9

**Published:** 2024-01-05

**Authors:** Tatsuaki Daimon, Atrayee Bhattacharya, Keyi Wang, Naoki Haratake, Ayako Nakashoji, Hiroki Ozawa, Yoshihiro Morimoto, Nami Yamashita, Takeo Kosaka, Mototsugu Oya, Donald W. Kufe

**Affiliations:** 1 Dana-Farber Cancer Institute, Harvard Medical School, Boston, MA USA; 2 Department of Gastroenterological Surgery, Kinan Hospital, Wakayama, Japan; 3 Breast Surgical Oncology, Breast Oncology Center, The Cancer Institute Hospital of the JFCR, Tokyo, Japan; 4 Department of Urology, Keio University School of Medicine, Tokyo, Japan

**Keywords:** Cancer stem cells, Small molecules

## Abstract

The oncogenic MUC1-C transmembrane protein is a critical effector of the cancer stem cell (CSC) state. Addiction to MUC1-C for self-renewal in the progression of human cancers has emphasized the need for development of anti-MUC1-C agents. However, there are presently no approved small molecules for targeting MUC1-C-dependent CSCs. In screening for small molecules, we identified salinomycin (SAL), an inducer of ferroptosis, as a potent inhibitor of MUC1-C signaling. We demonstrate that SAL suppresses MUC1-C expression by disrupting a NF-κB/MUC1-C auto-inductive circuit that is necessary for ferroptosis resistance. Our results show that SAL-induced MUC1-C suppression downregulates a MUC1-C→MYC pathway that activates genes encoding (i) glutathione-disulfide reductase (GSR), and (ii) the LDL receptor related protein 8 (LRP8), which inhibit ferroptosis by generating GSH and regulating selenium levels, respectively. GSR and LRP8 contribute to the function of glutathione peroxidase 4 (GPX4), an essential negative regulator of ferroptotic cell death. We demonstrate that targeting MUC1-C genetically or with the GO-203 peptide inhibitor suppresses GPX4 expression and GPX activity in association with the induction of ferroptosis. Studies of CSCs enriched by serial passage as tumorspheres further demonstrate that the effects of SAL are mediated by downregulation of MUC1-C and thereby overcoming resistance to ferroptosis. As confirmation of these results, rescue of MUC1-C downregulation with the MUC1-C cytoplasmic domain (i) reversed the suppression of GSR, LRP8 and GPX4 expression, and (ii) attenuated the induction of ferroptosis. These findings identify SAL as a unique small molecule inhibitor of MUC1-C signaling and demonstrate that MUC1-C is an important effector of resistance to ferroptosis.

## Introduction

The *MUCIN1* (*MUC1*) gene was identified based on its overexpression in breast and other human cancers [1, 2]. Subsequent work revealed that *MUC1* evolved in mammals to play a role in protecting barrier tissues from loss of homeostasis [3, 4]. These observations collectively supported the premise that the MUC1-mediated protective functions of epithelia are subverted in promoting cancer [3, 4]. Along these lines, *MUC1* encodes a transmembrane MUC1-C subunit that, when activated by stress, drives inflammatory, proliferative and remodeling responses associated with wound repair [4]. MUC1-C induces the epithelial-mesenchymal transition (EMT), epigenetic reprogramming and changes in chromatin architecture that are, in principle, reversible with restitution of homeostasis [4]. However, prolonged activation of MUC1-C in settings of chronic inflammation contributes to establishment of the cancer stem cell (CSC) state [3, 5]. Addiction to MUC1-C has thus been increasingly identified in CSCs from castration-resistant prostate cancer (CRPC), triple-negative breast cancer (TNBC) and other aggressive malignancies [5–10]. The CSC state confers resistance to anti-cancer treatment [11–16]. Consistent with this CSC capacity, MUC1-C drives resistance to pleotropic genotoxic and targeted agents mediated at least in part by an inflammatory memory response [9, 17–23]. These findings have supported the importance of MUC1-C as a target for eliminating CSCs, which is needed to improve clinical outcomes and achieve cures.

MUC1-C consists of a 58 aa extracellular domain that is being targeted with antibodies against conserved alpha-3 and alpha-4 helices [3, 4, 24, 25]. The MUC1-C cytoplasmic domain is a 72 aa intrinsically disordered peptide devoid of a kinase function [3, 4]. Accordingly, identification of small molecules that inhibit the MUC1-C cytoplasmic domain has been a challenge [26]. A cell-penetrating peptide, designated GO-203, was developed that blocks a CQC motif in the cytoplasmic domain necessary for MUC1-C homodimerization, nuclear import and oncogenic activity [3, 4, 27]. However, GO-203 administration was limited by a short circulating half-life and the need for delivery in a nanoparticle formulation [28]. Other strategies for targeting MUC1-C have included the development of CRISPR/cas vectors and anti-sense oligonucleotides [7, 8]. Nonetheless, to date, there has been limited success in developing small molecules that are effective in suppressing MUC1-C function. A small molecule screen has identified salinomycin (SAL) as one potential candidate, which is of particular interest in that SAL is an effective inhibitor of CSCs [29–32]. Of further interest, SAL induces ferroptosis of cancer cells [32], whereas MUC1-C attenuates the induction of ferroptosis [33]. MUC1-C drives expression of the xCT light chain of the cystine/glutamate transporter (system Xc-), which contributes to the production of glutathione (GSH) and is regarded as the most upstream regulator of ferroptotic cell death [33, 34]. The mechanisms by which SAL eliminates CSCs have been attributed to dysregulation of diverse pathways that include sequestration of iron in lysosomes, induction of autophagy and binding to nucleolin, among others [30, 32]. To our knowledge, there is no known link between MUC1-C and ferroptosis resistance in CSCs.

MUC1 expression is dysregulated across pan-cancers and is associated with poor clinical outcomes [35]; however, to date, there are no small molecules that target the oncogenic MUC1-C subunit. The present studies demonstrate that SAL is an effective small molecule inhibitor of MUC1-C signaling. SAL disrupts an NF-κB/MUC1-C auto-inductive circuit with the downregulation of MUC1-C expression. As a result, SAL inhibits a downstream MUC1-C→MYC pathway that increases chromatin accessibility and transcription of the *GSR* and *LRP8* genes. GSR and LRP8 regulate GPX4, a critical inhibitor of ferroptosis. Along these lines, we show that targeting MUC1-C suppresses GSR, LRP8 and GPX4 expression in association with inducing ferroptosis. CSCs are dependent on MUC1-C for self-renewal [5, 7–10, 23, 36]. Our results further demonstrate that CSCs are dependent on MUC1-C for resistance to ferroptosis.

## Results

### SAL downregulates MUC1-C expression in human cancer cells

**Fig. 1:**
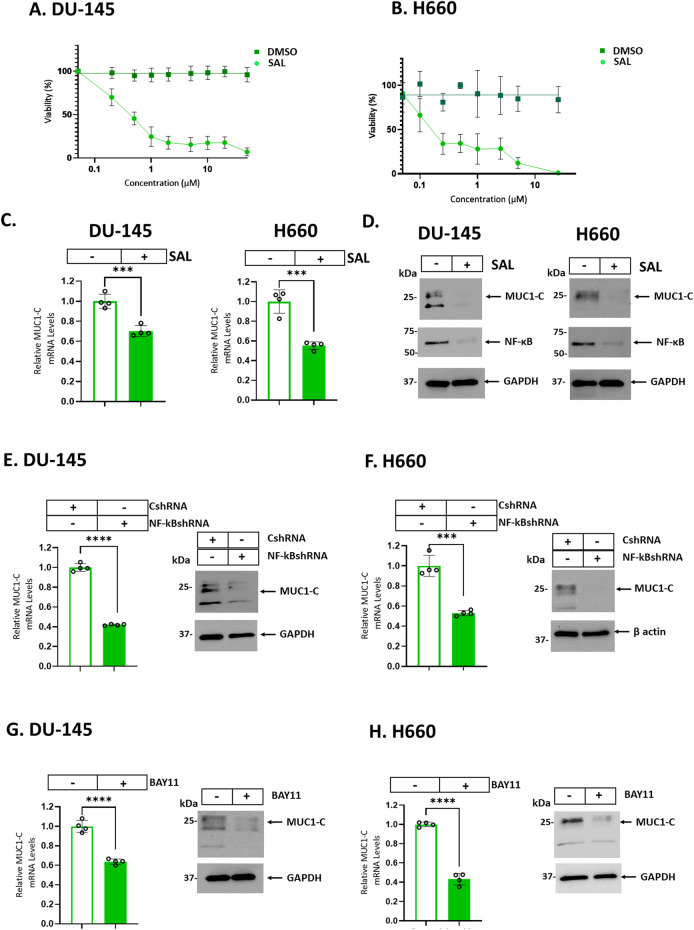
SAL suppresses the NF-κB/MUC1-C auto-inductive circuit. DU-145 (**A**) and H660 (**B**) cells were treated with vehicle (DMSO) or the indicated concentrations of SAL for 72 h. Viability was assessed by Alamar Blue staining. The results (mean ± SD of 4 determinations) are expressed as relative viability compared to untreated cells (assigned a value of 100%). **C**, **D** DU-145 and H660 cells treated with vehicle or 1 μM SAL for 24 h were analyzed for MUC1-C transcripts using primers listed in Supplementary Table S1 (**C**). The results (mean ± SD of 4 determinations) are expressed as relative MUC1-C mRNA levels compared to that obtained in vehicle-treated cells (assigned a value of 1). Lysates were immunoblotted with antibodies against the indicated proteins (**D**). DU-145 (**E**) and H660 (**F**) cells expressing a control CshRNA or NF-κBshRNA were analyzed for MUC1-C transcripts (left). The results (mean ± SD of 4 determinations) are expressed as relative MUC1-C mRNA levels compared to that obtained in CshRNA cells (assigned a value of 1). Lysates were immunoblotted with antibodies against the indicated proteins (right). DU-145 (**G**) and H660 (**H**) cells left untreated or treated with 10 μM BAY11-7082 for 24 h were analyzed for MUC1-C transcripts (left). The results (mean ± SD of 4 determinations) are expressed as relative MUC1-C mRNA levels compared to that obtained in untreated cells (assigned a value of 1). Lysates were immunoblotted with antibodies against the indicated proteins (right).

In investigating whether SAL affects MUC1-C expression, we found that treatment of DU-145 CRPC cells with increasing SAL concentrations suppresses their viability (IC50 = 0.45 μM) (Fig. 1A). Similar results were obtained with H660 NEPC cells; that is, a SAL concentration-dependent loss of viability (IC50 = 0.15 μM) (Fig. 1B). In concert with these effects, we found that SAL treatment of DU-145 and H660 cells is associated with decreases in MUC1-C transcripts (Fig. 1C, left and right). The *MUC1* gene is activated by proinflammatory transcription factors (TFs) that include NF-κB p65/RELA [37]. MUC1-C binds directly to RELA and contributes to the activation of NF-κB target genes, including *MUC1* itself in an auto-inductive circuit [37]. Of interest in this regard, SAL suppressed MUC1-C and NF-κB expression in DU-145 and H660 cells (Fig. 1D, left and right). In support of these results, silencing NF-κB decreased MUC1-C transcripts and protein (Fig. 1E, F). Moreover, treatment with the NF-κB inhibitor BAY11-7082 suppressed MUC1-C expression (Fig. 1G, H), confirming that SAL inhibits the NF-κB/MUC1-C auto-inductive pathway. We also found that SAL downregulates MUC1-C expression in BT-549 and MDA-MB-436 TNBC cells (Supplementary Figs. S1A, B), indicating that this response is not restricted to PC cells.

### SAL drives ferroptosis by downregulating MUC1-C expression

**Fig. 2:**
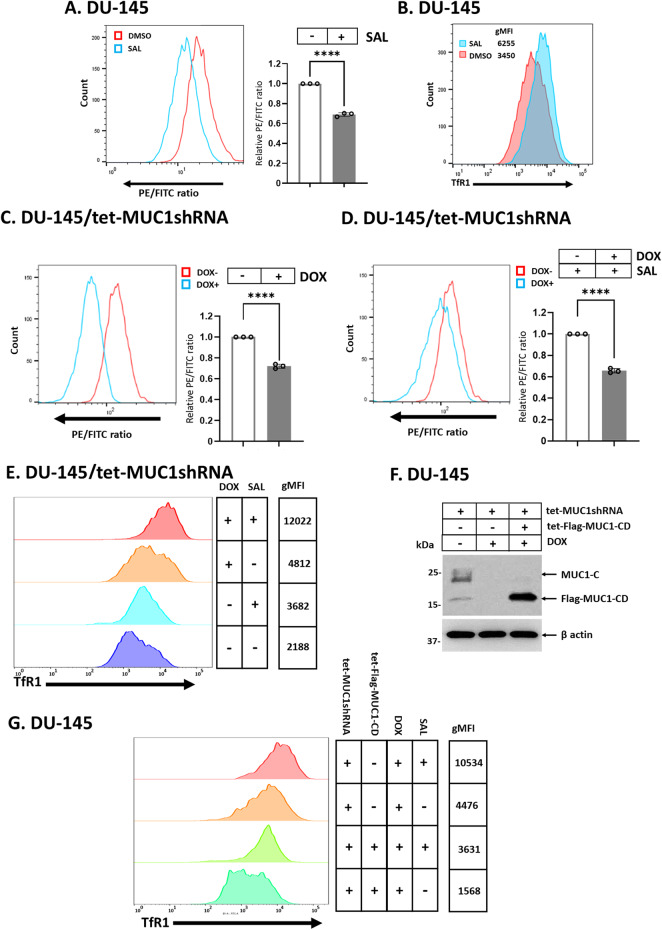
Silencing MUC1-C induces lipid peroxidation and enhances the effects of SAL treatment. **A** DU-145 cells treated with vehicle or 1 μM SAL for 24 h were analyzed for lipid peroxidation. Shown are histograms (left) and quantitation (mean ± SD of three determinations) (right) of the PE/FITC ratios. **B** DU-145 cells treated with vehicle or 1 μM SAL for 24 h were analyzed for TfR1 expression by flow cytometry. Listed are the gMFI values. **C** DU-145/tet-MUC1shRNA cells treated with vehicle or DOX for 7 days were analyzed for lipid peroxidation. Shown are histograms (left) and quantitation (mean ± SD of three determinations) (right) of the PE/FITC ratios. **D** DU-145/tet-MUC1shRNA cells treated with vehicle or DOX for 7 days and then incubated with 1 μM SAL for 24 h were analyzed for lipid peroxidation. Shown are histograms (left) and quantitation (mean ± SD of three determinations) (right) of the PE/FITC ratios. **E** DU-145/tet-MUC1shRNA cells treated with vehicle or DOX for 7 days and then incubated with 1 μM SAL for 24 h were analyzed for TfR1 expression by flow cytometry. Listed are the gMFI values. **F** Lysates from DU-145 cells expressing tet-MUC1shRNA and/or tet-MUC1-C/CD vectors treated with vehicle or DOX for 7 days were immunoblotted with antibodies against the indicated proteins. **G** DU-145 cells expressing tet-MUC1shRNA and/or tet-MUC1-C/CD vectors treated with vehicle or DOX for 7 days and then incubated with 1 μM SAL for 24 h were analyzed for TfR1 expression by flow cytometry. Listed are the gMFI values.

Treatment of DU-145 cells with SAL was associated with induction of lipid peroxidation as evidenced by staining with the ratiometric lipid peroxidase sensor (Fig. 2A). Consistent with these results, SAL increased cell surface expression of the transferrin receptor 1 (TfR1) marker of ferroptosis [38] (Fig. 2B). In assessing whether SAL-mediated downregulation of MUC1-C contributes to ferroptosis, we silenced MUC1-C in DU-145 and H660 cells (Supplementary Fig. S2A, B) and detected increases in lipid peroxidation (Fig. 2C; Supplementary Fig. S2C). In addition, silencing MUC1-C increased SAL-induced lipid peroxidation (Fig. 2D; Supplementary Fig. S2D) and TfR1 expression (Fig. 2E). As confirmation of these results, we rescued MUC1-C downregulation with expression of the MUC1-C cytoplasmic domain (tet-Flag-MUC1-C/CD), which unlike the endogenous *MUC1* gene, is under control by a tet-promoter (Fig. 2F). Importantly, rescue of MUC1-C expression suppressed the effects of silencing MUC1-C alone and in combination with SAL on the induction of ferroptosis (Fig. 2G) and cell death (Supplementary Fig. S2E). These results indicated that SAL induces ferroptosis, at least in large part, by suppressing MUC1-C expression.

### MUC1-C induces GSR expression and GSH production

**Fig. 3:**
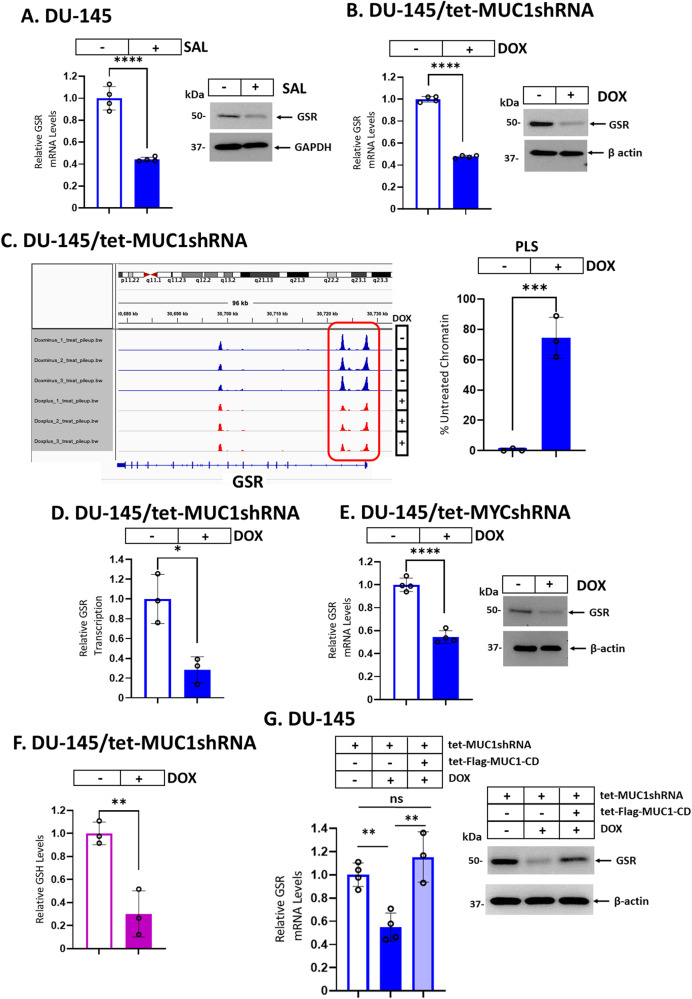
MUC1-C activates GSR expression by a MYC-dependent mechanism. DU-145 cells treated with vehicle or 1 μM SAL for 24 h (**A**) and DU-145/tet-MUC1shRNA cells treated with vehicle or DOX for 7 days (**B**) were analyzed for GSR transcripts (left). The results (mean ± SD of 4 determinations) are expressed as relative GSR mRNA levels compared to that obtained in vehicle-treated cells (assigned a value of 1). Lysates were immunoblotted with antibodies against the indicated proteins (right). **C** Genome browser snapshots of ATAC-seq data from the *GSR* gene in DU-145/tet-MUC1shRNA cells treated with vehicle or DOX for 7 days (left). Chromatin was analyzed for accessibility by nuclease digestion using primers listed in Supplementary Table S2 (right). The results (mean ± SD of 3 determinations) are expressed as % untreated chromatin. **D** DU-145/tet-MUC1shRNA cells treated with vehicle or DOX for 7 days were analyzed for *GSR* gene transcription. The results (mean ± SD of 3 determinations) are expressed as relative *GSR* transcription compared to that obtained in vehicle-treated cells (assigned a value of 1). **E** DU-145/tet-MYCshRNA cells treated with vehicle or DOX for 7 days were analyzed for GSR transcripts (left). The results (mean ± SD of 4 determinations) are expressed as relative GSR mRNA levels compared to that obtained in vehicle-treated cells (assigned a value of 1). Lysates were immunoblotted with antibodies against the indicated proteins (right). **F** DU-145/tet-MUC1shRNA cells treated with vehicle or DOX for 7 days were analyzed for GSH levels. The results (mean ± SD of 3 determinations) are expressed as relative GSH levels compared to that obtained in vehicle-treated cells (assigned a value of 1). **G** DU-145 cells expressing tet-MUC1shRNA and/or tet-Flag-MUC1-C/CD treated with vehicle or DOX for 7 days were analyzed for GSR transcripts (left). The results (mean ± SD of 4 determinations) are expressed as relative GSR mRNA levels compared to that obtained in vehicle-treated cells (assigned a value of 1). Lysates were immunoblotted with antibodies against the indicated proteins (right).

Glutathione-disulfide reductase (GSR) catalyzes the reduction of glutathione disulfide (GSSG) with NADPH as the electron donor. In this way, GSR generates GSH, which is necessary for conferring resistance to ferroptosis [39]. To our knowledge, there is no known relationship between SAL or MUC1-C with the regulation of GSR expression. We found that SAL treatment decreases GSR mRNA and protein levels (Fig. 3A). Moreover, silencing MUC1-C suppressed GSR expression (Fig. 3B; Supplementary Fig. S3A, B). In investigating how MUC1-C regulates GSR, ATAC-seq revealed dependence on MUC1-C for increasing chromatin accessibility of the *GSR* promoter region (Fig. 3C). In addition, silencing MUC1-C decreased *GSR* transcription (Fig. 3D). MYC binding motifs were identified in the *GSR* promoter, which was of interest in that the MUC1-C CQC motif binds directly to the MYC HLH-LZ domain and promotes the activation of MYC target genes [40]. Silencing MYC also suppressed GSR expression in concert with MUC1-C→MYC→GSR signaling (Fig. 3E). This pathway was further supported by the demonstration that silencing MUC1-C decreases GSH levels (Fig. 3F; Supplementary Fig. S3C). As confirmation of this MUC1-C dependence, rescuing MUC1-C silencing with MUC1-C/CD reestablished expression of GSR transcripts and protein (Fig. 3G).

### MUC1-C signaling regulates LRP8 and GPX4 expression

**Fig. 4:**
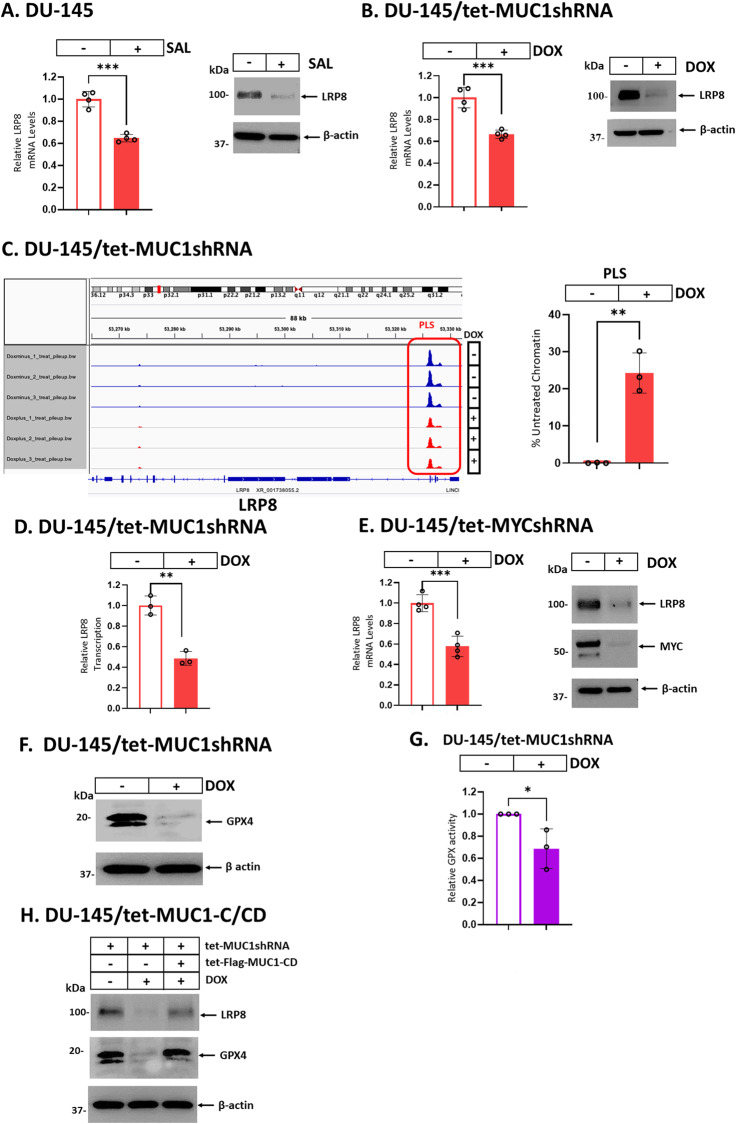
MUC1-C induces LRP8 and GPX4 expression. DU-145 cells treated with vehicle or 1 μM SAL for 24 h (**A**) and DU-145/tet-MUC1shRNA cells treated with vehicle or DOX for 7 days (**B**) were analyzed for LRP8 transcripts (left). The results (mean ± SD of 4 determinations) are expressed as relative LRP8 mRNA levels compared to that obtained in vehicle-treated cells (assigned a value of 1). Lysates were immunoblotted with antibodies against the indicated proteins (right). **C** Genome browser snapshots of ATAC-seq data from the *LRP8* gene in DU-145/tet-MUC1shRNA cells treated with vehicle or DOX for 7 days (left). Chromatin was analyzed for accessibility by nuclease digestion (right). The results (mean ± SD of 3 determinations) are expressed as % untreated chromatin. **D** DU-145/tet-MUC1shRNA cells treated with vehicle or DOX for 7 days were analyzed for *LRP8* gene transcription. The results (mean ± SD of 3 determinations) are expressed as relative *LRP8* transcription compared to that obtained in vehicle-treated cells (assigned a value of 1). **E** DU-145/tet-MYCshRNA cells treated with vehicle or DOX for 7 days were analyzed for LRP8 transcripts (left). The results (mean ± SD of 4 determinations) are expressed as relative LRP8 mRNA levels compared to that obtained in vehicle-treated cells (assigned a value of 1). Lysates were immunoblotted with antibodies against the indicated proteins (right). Lysates from DU-145/tet-MUC1shRNA cells treated with vehicle or DOX for 7 days were immunoblotted with antibodies against the indicated proteins (**F**) and analyzed for GPX activity (**G**). The results (mean ± SD of 3 determinations) are expressed as relative GPX activity compared to that obtained in vehicle-treated cells (assigned a value of 1). **H** Lysates from DU-145 cells expressing tet-MUC1shRNA and/or tet-Flag-MUC1-C/CD treated with vehicle or DOX for 7 days were immunoblotted with antibodies against the indicated proteins.

The LDL receptor related protein 8 (LRP8) regulates selenium levels and thereby ferroptosis in cancer cells [41]. There is no known involvement of SAL or MUC1-C in LRP8 regulation. We found that SAL treatment of DU-145 and H660 cells decreases LRP8 expression (Fig. 4A; Supplementary Fig. S4A). Silencing MUC1-C in these cells also downregulated LRP8 transcripts and protein (Fig. 4B; Supplementary Figs. S4B, C). As uncovered for *GSR*, silencing MUC1-C decreased *LRP8* gene chromatin accessibility (Fig. 4C) and transcription (Fig. 4D). We also identified MYC binding motifs in the *LRP8* promoter region and found that silencing MYC downregulates LRP8 expression (Fig. 4E). GPX4 is an essential negative regulator of ferroptotic cell death [42]. GSR has been linked to activation of GPX4 by maintaining GSH levels [42]. In addition, LRP8 regulates translation of the GPX4 protein [41]. Accordingly, we asked if MUC1-C also regulates GPX4 expression and found that silencing MUC1-C has little if any effect on GPX4 mRNA levels (Supplementary Fig. S4D), but decreases expression of the GPX4 protein (Fig. 4F; Supplementary Fig. S4E), in support of a post-transcriptional mechanism. Silencing MUC1-C was also associated with decreases in GPX activity (Fig. 4G; Supplementary Fig. S4F). Moreover, rescue of MUC1-C silencing with MUC1-C/CD reversed the suppression of LRP8 and GPX4 expression (Fig. 4H). These findings supported a model in which MUC1-C drives (i) GSR and LRP8 transcription by MYC-mediated activation, and thereby (ii) the regulation of GPX4 translation and activity.

### SAL-inhibited signaling is phenocopied by targeting MUC1-C with the GO-203 inhibitor

**Fig. 5:**
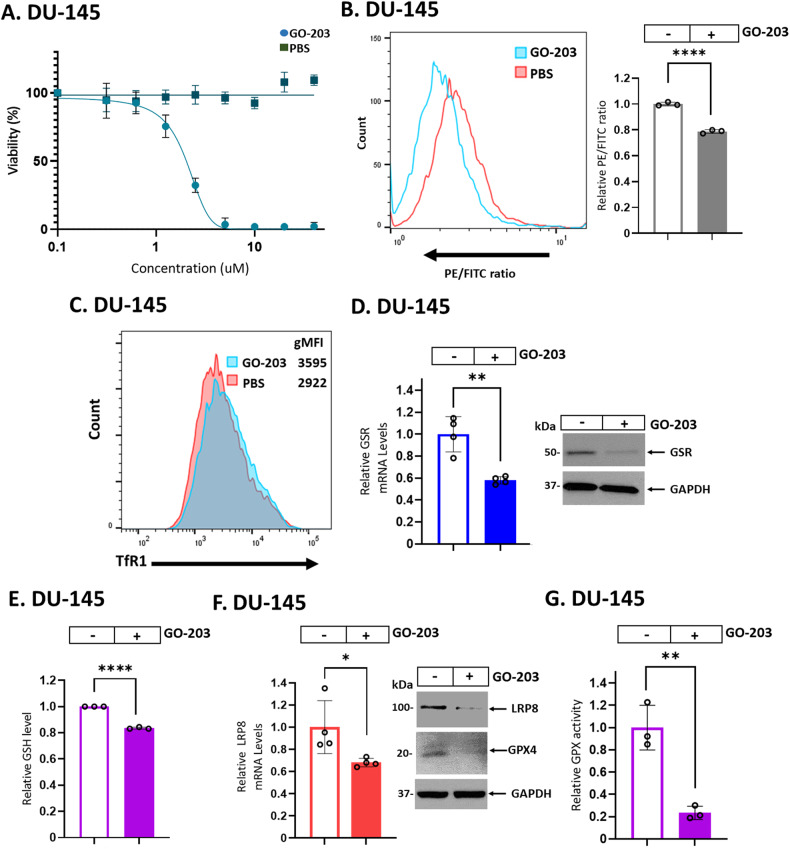
Targeting MUC1-C with GO-203 induces ferroptosis in association with suppression of GSR, LRP8 and GPX4 expression. **A** DU-145 cells were treated with PBS as a control vehicle or the indicated concentrations of GO-203 for 72 h. Viability was assessed by Alamar Blue staining. The results (mean ± SD of 4 determinations) are expressed as relative viability compared to untreated cells (assigned a value of 100%). **B** DU-145 cells left untreated or treated with 2 μM GO-203 for 24 h were analyzed for lipid peroxidation. Shown are histograms (left) and quantitation (mean ± SD of three determinations) (right) of the PE/FITC ratios. **C** DU-145 cells left untreated or treated with 2 μM GO-203 for 12 h were analyzed for cell surface TfR1 expression by flow cytometry. Listed are the gMFI values. **D** DU-145 cells left untreated or treated with 2 μM GO-203 for 24 h were analyzed for GSR transcripts (left). The results (mean ± SD of 4 determinations) are expressed as relative GSR mRNA levels compared to that obtained in untreated cells (assigned a value of 1). Lysates were immunoblotted with antibodies against the indicated proteins (right). **E** Lysates from DU-145 cells left untreated or treated with 2 μM GO-203 for 24 h were analyzed for GSH levels. The results (mean ± SD of 3 determinations) are expressed as relative GSH levels compared to that obtained in untreated cells (assigned a value of 1). **F** DU-145 cells left untreated or treated with 2 μM GO-203 for 8 h were analyzed for LRP8 transcripts (left). The results (mean ± SD of 4 determinations) are expressed as relative LRP8 mRNA levels compared to that obtained in untreated cells (assigned a value of 1). Lysates were immunoblotted with antibodies against the indicated proteins (right). **G** Lysates from DU-145 cells left untreated or treated with 2 μM GO-203 for 24 h were analyzed for GPX activity. The results (mean ± SD of 3 determinations) are expressed as relative GPX activity compared to that obtained in untreated cells (assigned a value of 1).

To confirm the effects of SAL-mediated MUC1-C downregulation on ferroptosis, cells were treated with the GO-203 inhibitor, which blocks the MUC1-C cytoplasmic domain CQC motif and thereby MUC1-C function [3, 4]. GO-203 treatment decreased DU-145 cell survival in a concentration-dependent manner (Fig. 5A), which was associated with induction of ferroptosis as evidenced by lipid peroxidation (Fig. 5B) and TfR1 expression (Fig. 5C). Similar results were obtained in H660 cells (Supplementary Fig. S5A, B), confirming that, like silencing MUC1-C, GO-203 induces ferroptosis. We also found that GO-203 treatment downregulates (i) GSR transcripts and protein (Fig. 5D; Supplementary Fig. S5C) and (ii) GSH levels (Fig. 5E; Supplementary Fig. S5D). Moreover, GO-203 decreased (ii) LRP8 and GPX4 expression (Fig. 5F; Supplementary Fig. S5E) and GPX activity (Fig. 5G; Supplementary Fig. S5F). These findings indicate that GO-203 phenocopies SAL-mediated MUC1-C downregulation and silencing MUC1-C genetically in inducing ferroptosis.

### Effects of SAL and targeting MUC1-C on CSC ferroptosis

**Fig. 6:**
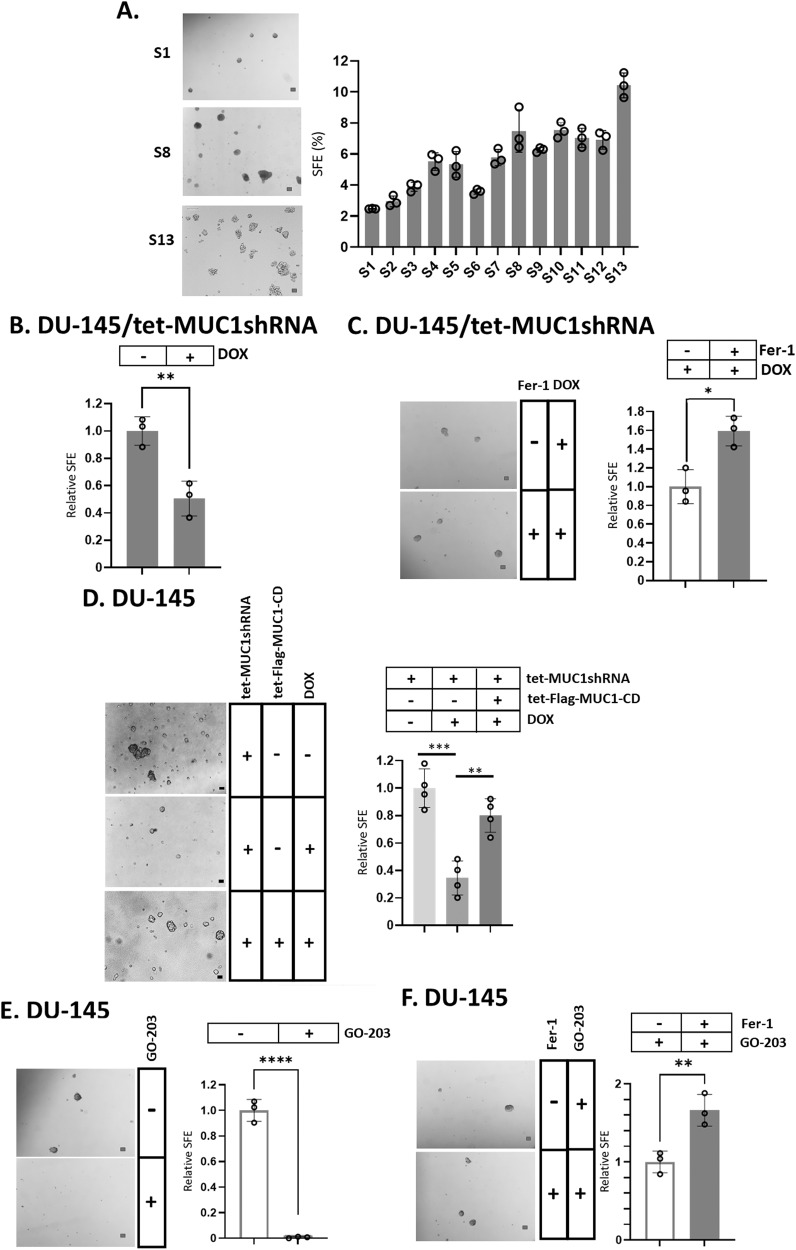
Enriched CSCs are dependent on MUC1-C for self-renewal capacity and resistance to ferroptosis. **A** DU-145 cells growing as monolayers were seeded in tumorsphere culture medium. After 10 days, the sphere 1 (S1) cells were isolated and reseeded for selection of S2 cells. Photomicrographs are shown for the serially passaged tumorspheres up to S13 (left). Scale bar: 100 μm. The sphere forming efficiency (SFE) was determined by the percentage of cells that formed tumorspheres as a function of the number of seeded cells. The results (mean ± SD of three determinations) are expressed as SFE (right). **B** DU-145/tet-MUC1shRNA CSCs treated with vehicle or DOX for 7 days were analyzed for tumorsphere formation. The results (mean ± SD of three determinations) are expressed as relative SFE compared to that obtained for vehicle treated cells (assigned a value of 1). **C** DU-145/tet-MUC1shRNA CSCs treated with vehicle or DOX in the absence and presence of 10 μM Fer-1 for 24 h were analyzed for tumorsphere formation. Photomicrographs are shown for the treated tumorspheres (left). The results (mean ± SD of three determinations) are expressed as relative SFE compared to that obtained in DOX alone treated cells (assigned a value of 1)(right). **D** DU-145/tet-MUC1shRNA CSCs without and with transfection of tet-Flag-MUC1-C/CD were treated with vehicle or DOX for 7 days. Photomicrographs are shown for the treated tumorspheres (left). The results (mean ± SD of three determinations) are expressed as relative SFE compared to that obtained in vehicle treated cells (assigned a value of 1) (right). **E** DU-145 CSCs treated with vehicle or 2 μM GO-203 for 24 h were analyzed for tumorsphere formation. Photomicrographs are shown for the treated tumorspheres (left). The results (mean ± SD of three determinations) are expressed as relative SFE compared to that obtained in vehicle treated cells (assigned a value of 1) (right) The results (mean ± SD of three determinations) are expressed as % SFE. **F** DU-145 CSCs treated with vehicle or 2 μM GO-203 in the absence and presence of 10 μM Fer-1 for 24 h were analyzed for tumorsphere formation. Photomicrographs are shown for the treated tumorspheres (left). The results (mean ± SD of three determinations) are expressed as relative SFE compared to that obtained in GO-203 alone treated cells (assigned a value of 1) (right).

CSCs from PC and other carcinomas are dependent on MUC1-C for self-renewal capacity and tumorigenicity [4, 6–8, 36, 43, 44]. However, it is not known if this dependence is a function of suppressing ferroptosis. Accordingly, we enriched DU-145 CSCs by serial passage (S1 to S13) of tumorspheres, which was associated with progressive increases in sphere forming efficiency (SFE) (Fig. 6A). Treatment of the enriched CSCs with SAL downregulated expression of MUC1-C and the PC stem cell marker CD133 [45] (Supplementary Fig. S6A). SAL treatment of the CSCs also decreased self-renewal capacity (Supplementary Fig. S6B), which was abrogated by the ferroptosis inhibitor Ferrostatin-1 (Fer-1) (Supplementary Fig. S6C). Consistent with these results and SAL-induced downregulation of MUC1-C, we found that silencing MUC1-C in DU-145 CSCs similarly decreases self-renewal (Fig. 6B) and that Fer-1 blocks this response (Fig. 6C). Importantly, rescue of MUC1-C silencing with MUC1-C/CD reversed suppression of self-renewal (Fig. 6D). As confirmation of these results, we found that treatment with GO-203 is associated with suppression of NF-κB (Supplementary Fig. S6D) and that NF-κB is necessary for tumorsphere formation (Supplementary Fig. S6E). Targeting CSCs with GO-203 was also associated with loss of self-renewal capacity (Fig. 6E) and induction of ferroptosis (Fig. 6F), indicating that enriched CSCs are dependent on MUC1-C for self-renewal and ferroptosis resistance.

### SAL downregulates MUC1-C expression in association with suppression of tumorigenicity

**Fig. 7:**
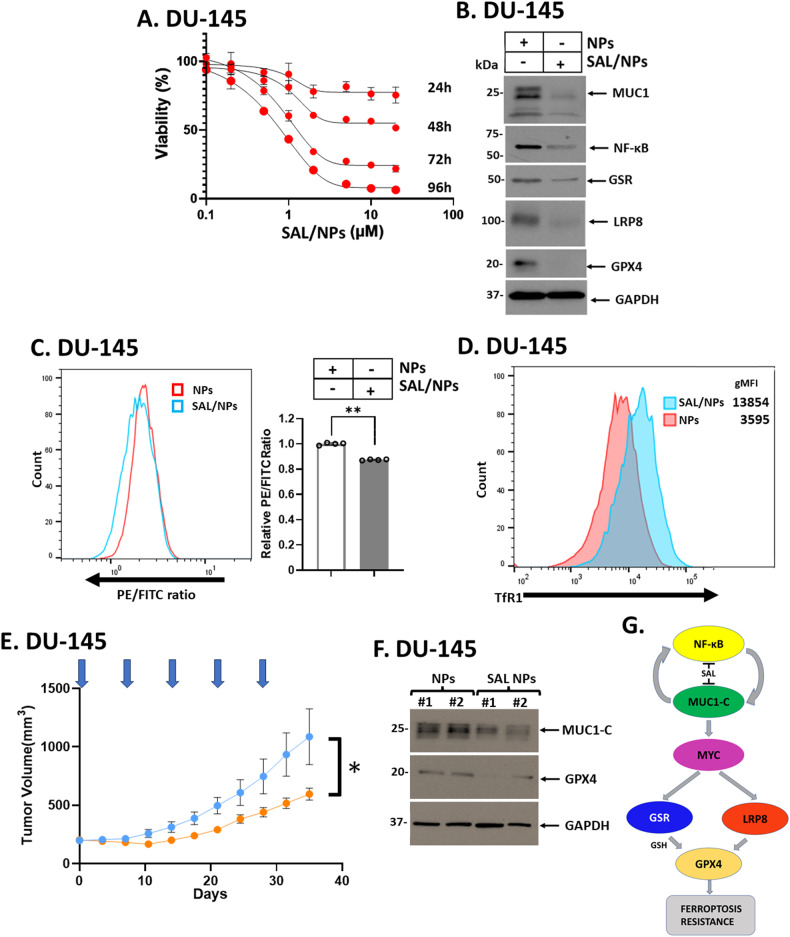
MUC1-C is a target of SAL/NPs for inhibition of tumorigenicity. **A** DU-145 cells were treated with SAL/NPs at the indicated concentrations of SAL for 24–96 h. Viability was assessed by Alamar Blue staining. The results (mean ± SD of 4 determinations) are expressed as relative viability compared to untreated cells (assigned a value of 100%). **B** Lysates from DU-145 cells treated with SAL/NPs or an equivalent amount of empty NPs for 24 h were immunoblotted with antibodies against the indicated proteins. **C** DU-145 cells treated with SAL/NPs or an equivalent amount of empty NPs for 24 h were analyzed for lipid peroxidation. Shown are histograms (left) and quantitation (mean ± SD of three determinations) (right) of the PE/FITC ratios. **D** DU-145 cells treated with SAL/NPs or an equivalent amount of empty NPs for 24 h were analyzed for TfR1 expression by flow cytometry. Listed are the gMFI values. **E** Six-week old nude mice were injected subcutaneously in the flank with 1 × 10^7^ DU-145 cells. Mice pair-matched into two groups of 6 mice each when tumors reached 150–200 mm^3^ were treated with SAL/NPs or empty NPs each week x 5 weeks. Tumor volumes are expressed as the mean ± SEM for six mice. **F** Lysates of tumors harvested on day 35 were immunoblotted with antibodies against the indicated proteins. **G** Schema depicting the effects of SAL on inhibiting MUC1-C-driven ferroptosis resistance. MUC1-C interacts directly with NF-κB and contributes to the activation of NF-κB target genes, including *MUC1* in an auto-inductive loop. SAL inhibits this inflammatory circuit by suppressing NF-κB and MUC1-C expression. The MUC1-C cytoplasmic domain also binds directly to MYC and activates MYC target genes. Our results demonstrate that this MUC1-C→MYC pathway is necessary for increasing chromatin accessibility of the *GSR* and *LRP8* promoter regions and activation of these genes. The dependency on MUC1-C for induction of GSR and LRP8 expression was confirmed by rescuing MUC1-C silencing with expression of the MUC1-C cytoplasmic domain which includes the CQC motif that binds to MYC. Moreover, this MUC1-C dependency was extended by the demonstration that the MUC1-C inhibitor GO-203, which blocks the CQC motif, similarly suppresses GSR and LRP8 expression. GPX4 is an essential negative regulator of ferroptosis. GSR regulates GSH levels necessary for GPX4 activity. In addition, LRP8 contributes to translation of the GPX4 protein. Consistent with dependence on MUC1-C for GSR and LRP8 expression, targeting MUC1-C genetically and pharmacologically with SAL or GO-203 downregulated GPX4 levels. Rescue of MUC1-C silencing with the MUC1-C cytoplasmic domain further confirmed that MUC1-C is necessary for GPX4 expression and ferroptosis resistance. These findings support a model in which SAL-induced ferroptosis is conferred, at least in large part, by downregulation of MUC1-C and thereby suppression of GSR, LRP8 and GPX4.

Delivery of SAL in humans has been challenged by (i) highly lipophilic properties, and (ii) associated muscular and neurotoxicity [46, 47]. In addressing these challenges, the encapsulation of SAL in polymeric nanoparticles and other nanoformulations has advanced SAL treatment with enhanced therapeutic indices [47]. To extend the present work, we treated DU-145 cells with SAL/polymeric nanoparticles (SAL/NPs) and found a concentration- and time-dependent inhibition of viability (Fig. 7A). By contrast, empty polymeric nanoparticles (NPs) had little if any effect (Supplementary Fig. S7A). As shown for SAL, treatment with SAL/NPs suppressed MUC1-C and NF-κB expression (Fig. 7B). SAL/NPs were also effective in downregulating (i) GSR, LRP8 and GPX4 levels (Fig. 7B), and (ii) inducing ferroptosis (Fig. 7C, D). Consistent with these results, treatment of DU-145 tumor xenografts with SAL/NPs, but not empty NPs, suppressed tumorigenicity in the absence of body weight loss or other overt toxicities (Fig. 7E; Supplementary Fig. S7B). Analysis of tumors from SAL/NP-treated mice further demonstrated decreases in expression of MUC1-C and the essential GPX4 negative regulator of ferroptosis (Fig. 7F). These findings confirmed that MUC1-C is a target of SAL in tumors.

## Discussion

MUC1-C evolved in mammals to protect barrier tissues from loss of homeostasis [4]. MUC1-C activates inflammatory, proliferative and remodeling responses in resident SCs that are integral to wound repair [4, 48]. However, prolonged activation of MUC1-C in settings of chronic inflammation and damage has the capacity to promote the progression of resident SCs to CSCs [4]. In support of this premise, MUC1-C drives lineage plasticity and self-renewal in CSCs derived from epithelial cells in the prostate, breast, respiratory tract and other organs with protective barrier functions [4]. As selected examples, studies of CRPC/NEPC [6, 10], TNBC [5], small cell lung cancer (SCLC) [8] and Merkel Cell Carcinoma (MCC) [7] cells have demonstrated dependence on MUC1-C for self-renewal capacity and tumorigenicity. The present work demonstrates that MUC1-C addiction of enriched CSCs involves resistance to ferroptosis. Screening for selective inhibitors of CSCs identified SAL as the lead candidate based on potent activity in suppressing tumorsphere formation and tumor growth [29]. In our search for small molecule inhibitors of MUC1-C signaling, we also identified SAL, which was of particular interest given the more recent evidence that CSCs are dependent on MUC1-C expression [4]. We found that SAL inhibits an inflammatory pathway in which NF-κB induces MUC1-C expression and, in turn, MUC1-C binds directly to NF-κB in regulating NF-κB target genes, including *MUC1* itself (Fig. 7G) [4, 37]. The effects of SAL on CSCs have been attributed to (i) inhibition of the WNT/β-catenin and the ERK signaling pathways [49, 50], (ii) induction of autophagy [51], and disruption of redox balance [52]. How SAL induces these pleotropic effects have remained largely unclear. Nonetheless, of potential relevance is that the MUC1-C cytoplasmic domain (MUC1-C/CD) functions as a scaffold in regulating the WNT/β-catenin and ERK pathways, as well as ROS-induced cell death [3, 4, 48].

Previous work demonstrated that MUC1-C interacts with xCT, which has been linked to the regulation of ferroptosis [33]. The present findings that SAL downregulates MUC1-C has now identified a previously unrecognized role for MUC1-C in conferring ferroptosis resistance. Our results demonstrate that SAL suppresses key effectors of ferroptosis resistance by MUC1-C dependent mechanisms (Fig. 7G). The MUC1-C cytoplasmic domain interacts directly with MYC to regulate MYC target genes that encode effectors of epigenetic reprogramming and chromatin remodeling. In this way, MUC1-C→MYC signaling drives dedifferentiation and the CSC state [40, 53]. Our results extend involvement of the MUC1-C→MYC pathway to the induction of GSR expression, which contributes to GSH production and GPX4 activation (Fig. 7G) [39]. We also found that the MUC1-C→MYC pathway activates expression of LRP8, an effector of GPX4 expression and activity (Fig. 7G) [41]. ATAC-seq studies demonstrated that MUC1-C increases chromatin accessibility of the *GSR* and *LRP8* genes in association with activation of their transcription. MUC1-C regulates components of the SWI/SNF esBAF and PBAF chromatin remodeling complexes that are necessary for expression stemness genes, such as NOTCH1, and others that encode effectors of redox balance needed for maintaining the CSC state [43, 54]. Along these lines, MUC1-C induces differentially accessible regions (DARs) across the genomes of CSCs that align with promoter and enhancer signatures in genes regulated by the JUN/AP-1 family of TFs [55]. Additional studies will be needed to determine if the MUC1-C-induced DARs in the *GSR* and *LRP8* promoters involve recruitment of AP-1 TFs in that those regions include AP-1 binding motifs. In contrast to GSR and LRP8, we found that MUC1-C has no apparent effect on chromatin accessibility and transcription of the *GPX4* gene. Rather, MUC1-C regulates GPX4 levels by a post-transcriptional mechanism, which may be attributable to the effects of MUC1-C on GSR and LRP8 expression and thereby the downstream regulation of GPX4 (Fig. 7G).

SAL kills CSCs by sequestering iron in lysosomes and inducing ferroptosis [30]. Our results demonstrate that silencing MUC1-C is sufficient for inducing ferroptosis as evidenced by induction of lipid peroxidation and TfR1 cell surface expression. Similar observations were obtained when targeting MUC1-C with the GO-203 inhibitor, confirming that MUC1-C is required for ferroptosis resistance. We therefore conclude that SAL induces ferroptosis, at least in part, by downregulating MUC1-C and, in turn, GSR and LRP8 expression (Fig. 7G). In support of this conclusion, rescue of MUC1-C downregulation with MUC1-C/CD reversed the suppression of GSR, LRP8 and GPX4 in association with restitution of ferroptosis resistance. MUC1-C/CD is an intrinsically disordered protein that interacts with diverse kinases and functions as a scaffold for nodes that integrate signaling pathways [4]. Therefore, we do not exclude the possibility that MUC1-C drives ferroptosis resistance by mechanisms other than those identified in the present work. Of importance is that, in addition to being necessary for CSC self-renewal, MUC1-C also drives resistance of enriched CSCs to ferroptosis (Fig. 7G). The CSC state confers unresponsiveness to anti-cancer therapies and poor clinical outcomes [11–16]. In this context, MUC1-C dependencies extend to other CSC functions that include resistance to genotoxic drugs [17–19] and targeted agents, such as tamoxifen, trastuzumab, afatinib, vemurafenib and osimertinib [9, 20–23]. Moreover, targeting MUC1-C sensitizes resistant cells to these agents in support of reversing the responsible mechanisms. These and the present findings collectively support CSC dependency on MUC1-C for self-renewal and memory responses that confer drug and ferroptosis resistance [3, 4, 18–20, 22, 23, 48]. Our findings further support the development of SAL for targeting MUC1-C-expressing CSCs by encapsulating SAL in NPs conjugated to anti-MUC1-C antibodies [25].

## Materials and methods

### Cell culture

DU-145 CRPC cells (ATCC) and NCI-H660 NEPC cells (ATCC) were cultured in RPMI1640 medium (Thermo Fisher Scientific, Waltham, MA, USA) supplemented with 10% FBS. BT-549 TNBC cells (ATCC) were cultured in RPMI1640 medium containing 10% FBS and 10 μg/mL insulin. MDA-MB-468 TNBC cells were cultured in Leibovitz’s L-15 Medium (Thermo Fisher Scientific) supplemented with 10% FBS. Cells were treated with the MUC1-C inhibitor GO-203 [3, 4, 48], salinomycin (S8129, SelleckChem, Houston, TX, USA), BAY11-7082 (SelleckChem) and salinomycin encapsulated in polymeric nanoparticles (SAL/NPs, HSB-1216; HillstreamBiopharma, Bridgewater, NJ, USA). Cell viability was assessed using the Alamar Blue assay (Thermo Scientific, Rockford, IL, USA) in sextuplicate wells. The IC50 value was determined by nonlinear regression of the dose–response data using Prism 9.0 (GraphPad Software). Authentication of the cells was performed by short tandem repeat (STR) analysis. Cells were monitored for mycoplasma contamination using the MycoAlert Mycoplasma Detection Kit (Lonza, Rockland, MA, USA). Cells were maintained for 3 months for performing experiments.

### Gene silencing and rescue vectors

MUC1shRNA (MISSION shRNA TRCN0000122938; Sigma, St. Louis, MO, USA), MYCshRNA (MISSION shRNA TRCN0000039642; Sigma) or a control scrambled shRNA (CshRNA; Sigma) was inserted into the pLKO-tet-puro vector (Plasmid #21915; Addgene, Cambridge, MA, USA) as described [8]. The MUC1shRNA#2 (MISSION shRNA TRCN0000430218) was produced in HEK293T cells as described [56]. Flag-tagged MUC1-CD [57] was inserted into pInducer20 (Plasmid #44012, Addgene) [58]. Cells transduced with the vectors were selected for growth in 1–2 μg/ml puromycin. Cells were treated with 0.1% DMSO as the vehicle control or 500 ng/ml DOX (Millipore Sigma, Burlington, MA, USA).

### qRT-PCR

Total cellular RNA was isolated using Trizol reagent (Thermo Fisher Scientific). cDNAs were synthesized using the High Capacity cDNA Reverse Transcription Kit (Applied Biosystems, Grand Island, NY, USA). The cDNA samples were amplified using the Power SYBR Green PCR Master Mix (Applied Biosystems) and the CFX96 Real-Time PCR System (BIO-RAD, Hercules, CA, USA) as described [7]. Primers used for qRT-PCR are listed in Supplementary Table S1.

### Immunoblot analysis

Total lysates prepared from non-confluent cells were subjected to immunoblot analysis using anti-MUC1-C (HM-1630-P1ABX, 1:1000 dilution; Thermo Fisher Scientific), anti-β-actin (A5441, 1:5000 dilution; Sigma-Aldrich), anti-GAPDH (#2118, 1:1000; CST), anti-NF-κB p65 (ab32536, 1:1000 dilution, Abcam, Cambridge, MA, USA), anti-GPX4 (#52455, 1:1000 dilution, CST), anti-GSR (18257-1-AP, 1:2000 dilution; PROTEINTECH, Rosemont, IL, USA), anti-LRP8 (NB100-2216, 1:1000 dilution; Novus Biologicals, Centennial, CO, USA) and anti-CD133 (#5860, 1:1000 dilution, CST).

### Lipid peroxidation assay

Ratiometric measurement of lipid peroxidation was performed using the Lipid Peroxidation Assay Kit (ab243377; Abcam) according to the manufacturer’s instructions. Cells were analyzed by MACSQuant Analyzer 10 Flow Cytometer (Miltenyi Biotec, Waltham, MA, USA). Measurement of PE/FITC ratio was performed with FlowJo v10.6.2 (BD Biosciences, Franklin Lakes, NJ, USA) software.

### Flow cytometry

Cells were blocked with 1% BSA/PBS for 20 minutes on ice. After washing with ice cold PBS, cells were incubated with anti-TfR1 (CD71) antibody (MABC1765, clone 3F3-FMA, 1:100 dilution; Millipore Sigma) or an IgG1 isotype control antibody (MOPC-21, 1:100 dilution; BioLegend, San Diego, CA, USA) for 40 min on ice. FITC-conjugated goat F(ab)2 anti-mouse immunoglobulin was used as the secondary reagent (115-096-146, 1:100 dilution, Jackson ImmunoResearch, West Grove, PA, USA). Dead cells were stained with eBioscience 7-AAD viability staining solution (00-6993-50, Invitrogen). Cell death rates were measured using propidium iodide (PI) (Thermo Scientific). Cells were analyzed by MACSQuant Analyzer 10 Flow Cytometer (Miltenyi Biotec). Measurement of geometric MFI (gMFI) was performed with FlowJo v10.6.2 (BD Biosciences) software.

### Gene transcription assays

Newly synthesized RNA transcripts were detected using the Click-iT Nascent RNA Capture Kit (C10365; Thermo Fisher Scientific) according to the manufacturer’s instructions. The captured transcripts were analyzed using qRT-PCR.

### ATAC-seq

ATAC-seq libraries were generated from three biologically independent replicates per condition as described [55]. Chromatin accessibility was assessed using Integrative Genomics Viewer (IGV_2.13.0).

### Chromatin accessibility assay

DNase I chromatin accessibility assays were performed as described [55]. The DNA was purified and amplified by qPCR using the primers listed in Supplementary Table S2.

### Measurement of GSH levels

GSH (GSH-Glo Glutathione Assay, V6911; Promega, Madison, WI, USA) levels were determined according to the manufacturer’s instructions. Luminescence intensity was detected using FLUOstar Omega plate reader (BMG LABTECH, Cary, NC, USA).

### Measurement of GPX activity levels

Assays for GPX activity were performed using the Glutathione Peroxidase Assay Kit (MAK437; Millipore Sigma) according to the manufacturer’s instructions. GPX activity was measured using FLUOstar Omega plate reader (BMG LABTECH).

### Tumorsphere formation assays

Cells (2–6 × 10^3^) were seeded per well in 6-well ultra-low attachment culture plates (Corning Life Sciences, Corning, NY, USA) in DMEM/F12 50/50 medium (Corning Life Sciences) with 20 ng/ml EGF (Millipore Sigma), 20 ng/ml bFGF (Millipore Sigma) and 1% B27 supplement (Gibco). Tumorspheres were counted under an inverted microscope in triplicate wells.

### Mouse tumor xenograft studies

Six- to 8-week old nude mice (Taconic Farms, Germantown, NY, USA) were injected subcutaneously in the flank with 1 × 10^7^ DU-145 cells in 100μl of a 1:1 solution of medium and Matrigel (BD Biosciences). When the mean tumor volume reached 150–200 mm^3^, mice were pair-matched into groups of 6 mice each. Mice were treated intraperitoneally with SAL/NPs (5 mg SAL/kg) or an equivalent amount of empty NPS each week × 5 weeks. Unblinded tumor measurements as calculated by the formula: (width)^2^ × length/2 and body weights were recorded twice each week. Mice were sacrificed when control tumors reached >2000 mm^3^. The resource equation method was used to determine the minimum number of mice for achieving significance [59]. These studies were conducted in accordance with ethical regulations required for approval by the Dana-Farber Cancer Institute Animal Care and Use Committee (IACUC) under protocol 03-029.

### Statistics

Each experiment was performed at least three times. Data are expressed as the mean ± SD. The unpaired Mann–Whitney U test was used to determine differences between means of groups. A *p*-value of <0.05 denoted by an asterisk (*) was considered statistically significant.

### Supplementary information

supplementary figures and tables

Original Data File

## Data Availability

The accession number for the ATAC-seq data is GEO Submission GSE180599.
