# Supplementary material for: MUC1-C is a target of salinomycin in inducing ferroptosis of cancer stem cells
Source: Cell Death Discov. 2024 Jan 5;10:9. doi: 10.1038/s41420-023-01772-9 (PMC10770371; doi:10.1038/s41420-023-01772-9)
Supplement: Supplementary file 1 — supplementary figures and tables [file 41420_2023_1772_MOESM1_ESM.pdf]

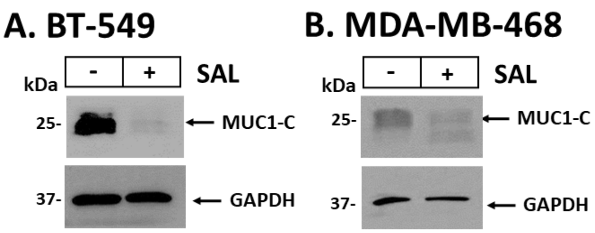

Supplementary Figure S1. SAL downregulates MUC1-C expression in TNBC cells. A and B. Lysates from BT-549 (**A**) and MDA-MB-468 (**B**) cells treated with vehicle or 1 μM SAL for 48 hours were immunoblotted with antibodies against the indicated proteins.

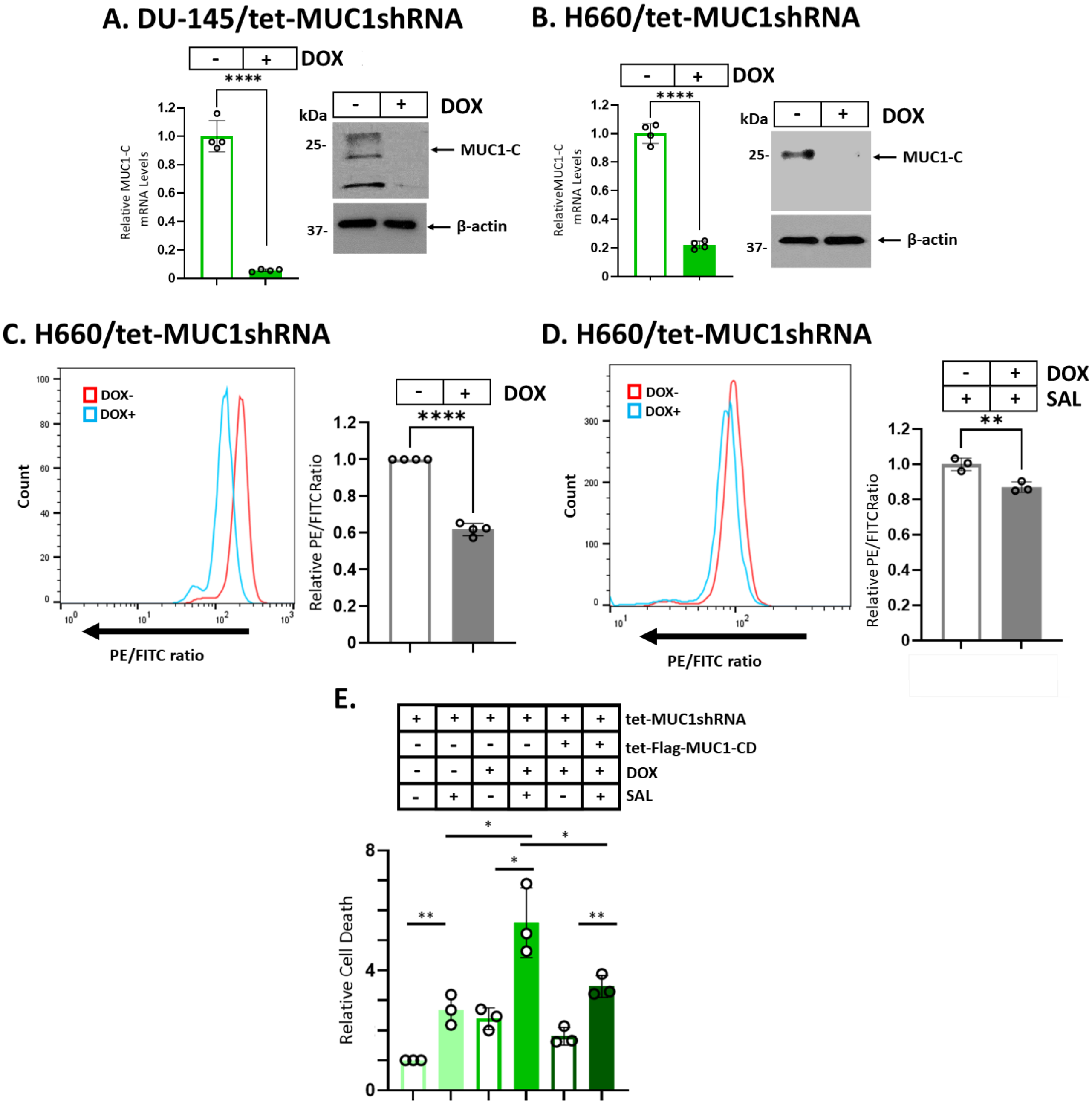

Supplementary Figure S2. Effects of silencing MUC1-C on induction of ferroptosis. A and B. DU-145/tet-MUC1shRNA (**A**) and H660/tet-MUC1shRNA (**B**) cells treated with vehicle or DOX for 7 days were analyzed for MUC1-C mRNA levels by qRT-PCR using primers listed in Supplementary Table S1. The results (mean±SD of four determinations) are expressed as relative levels compared to that obtained for vehicle-treated cells (assigned a value of 1) (left). Lysates were immunoblotted with antibodies against the indicated proteins (right). **C.** H660/tet-MUC1shRNA cells treated with vehicle or DOX for 7 days were analyzed for lipid peroxidation. Shown are histograms (left) and quantitation (mean±SD of three determinations) (right) of the PE/FITC ratios. **D.** H660/tet-MUC1shRNA cells treated with vehicle or DOX for 7 days and then incubated with 1 uM SAL for 24 hours were analyzed for

lipid peroxidation. Shown are histograms (left) and quantitation (mean±SD of three determinations) (right) of the PE/FITC ratios. **E.** DU-145 cells expressing tet-MUC1shRNA and tet-MUC1-C/CD vectors were treated with vehicle or DOX for 5 days and then 1 μM SAL for an additional 2 days were analyzed for cell death by PI staining. The results (mean±SD of four determinations) are expressed as relative cell death compared to that obtained for vehicle-treated cells (assigned a value of 1).

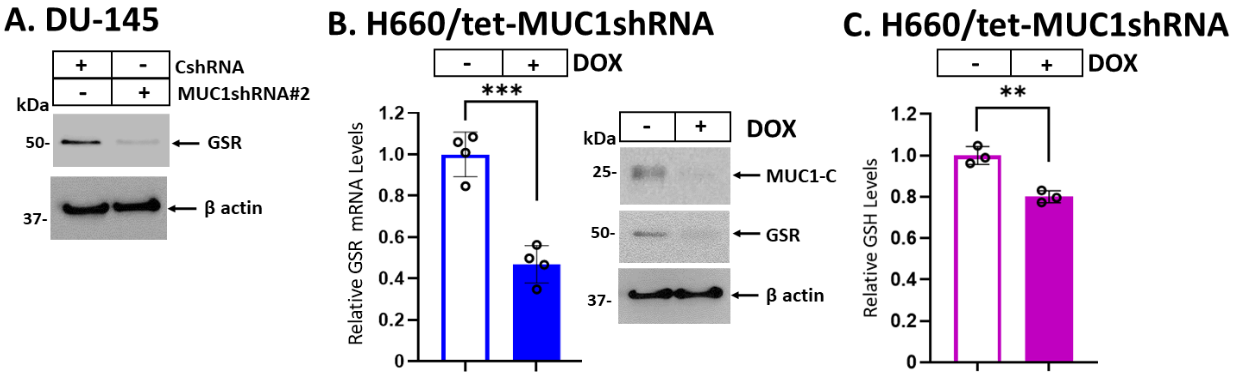

Supplementary Figure S3. Silencing MUC1-C with MUC1shRNA#2 suppresses GSR expression. A. DU-145/CshRNA and DU-145/MUC1shRNA#2 were analyzed for GSR transcripts (left). The results (mean±SD of 4 determinations) are expressed as relative GSR mRNA levels compared to that obtained in CshRNA cells (assigned a value of 1). Lysates were immunoblotted with antibodies against the indicated proteins (right). **B.** H660/tet-MUC1shRNA cells treated with vehicle or DOX for 7 days were analyzed for GSR transcripts (left). The results (mean±SD of 4 determinations) are expressed as relative GSR mRNA levels compared to that obtained in vehicle-treated cells (assigned a value of 1). Lysates were immunoblotted with antibodies against the indicated proteins (right). **C.** H660/tet-MUC1shRNA cells treated with vehicle or DOX for 7 days were analyzed for GSH levels. The results (mean±SD of 3 determinations) are expressed as relative GSH levels compared to that obtained in vehicle-treated cells (assigned a value of 1).

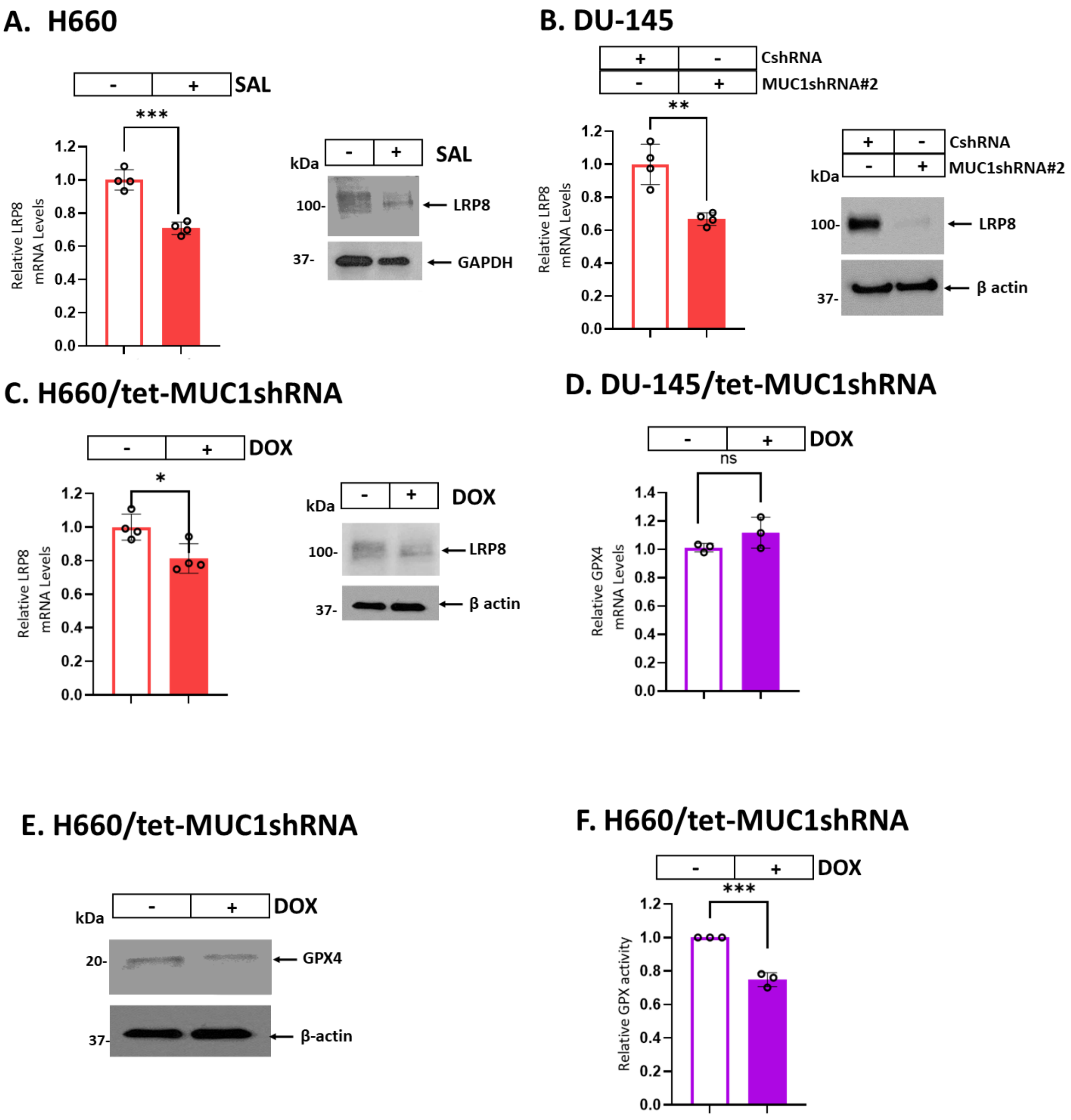

Supplementary Figure S4. Effects of MUC1-C on LRP8 and GPX4 expression. A. H660 cells treated with vehicle or 1 µM SAL for 24 hours were analyzed for LRP8 transcripts (left). The results (mean±SD of 4 determinations) are expressed as relative LRP8 mRNA levels compared to that obtained in vehicle-treated cells (assigned a value of 1). Lysates were immunoblotted with antibodies against the indicated proteins (right). **B.** DU-145/CshRNA and DU-145/MUC1shRNA#2 were analyzed for LRP8 transcripts (left). The results (mean±SD of 4 determinations) are expressed as relative GSR mRNA levels compared to that obtained in CshRNA cells (assigned a value of 1). Lysates were immunoblotted with antibodies against the indicated proteins (right). **C.** H660/tet-MUC1shRNA cells treated with vehicle or DOX for 7 days were analyzed for LRP8 transcripts (left). The results (mean±SD of 4 determinations) are expressed as relative LRP8 mRNA levels compared to that obtained in

vehicle-treated cells (assigned a value of 1). Lysates were immunoblotted with antibodies against the indicated proteins (right). **D.** DU-145/tet-MUC1shRNA cells treated with vehicle or DOX for 7 days were analyzed for GPX4 transcripts. The results (mean±SD of 4 determinations) are expressed as relative levels compared to that obtained in vehicle-treated cells (assigned a value of 1). **E and F.** Lysates from H660/tet-MUC1shRNA cells treated with vehicle or DOX for 7 days were immunoblotted with antibodies against the indicated proteins (**E**) and analyzed for GPX activity (**F**). The results (mean±SD of 3 determinations) are expressed as relative GPX activity compared to that obtained in vehicle-treated cells (assigned a value of 1).

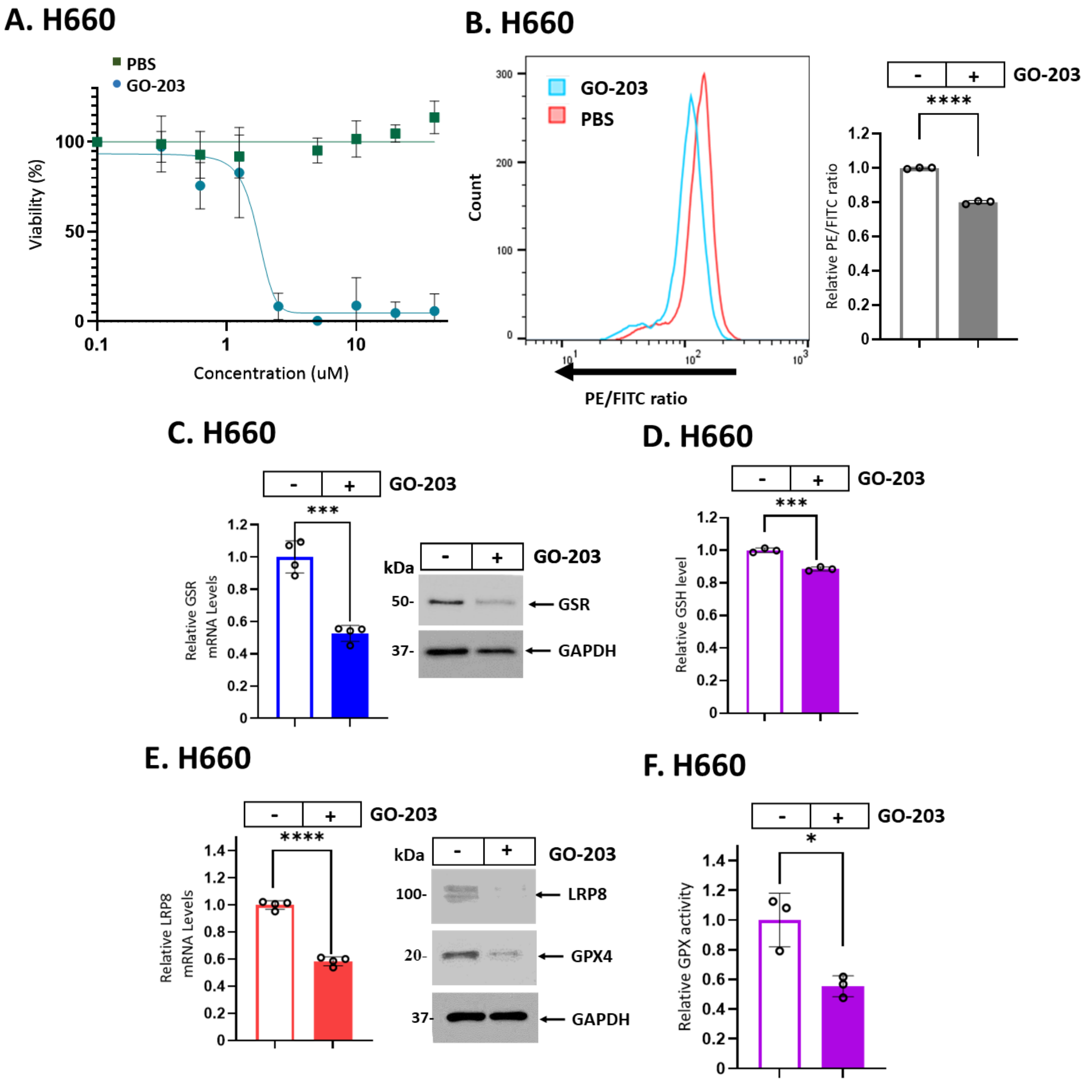

Supplementary Figure S5. Effects of targeting MUC1-C with GO-203 in H660 cells. A. H660 cells were treated with vehicle or the indicated concentrations of GO-203 for 72 hours. Viability was assessed by Alamar Blue staining. The results (mean±SD of 3 determinations) are expressed as relative viability compared to untreated cells (assigned a value of 100%). **B.** H660 cells left untreated or treated with 2 μM GO-203 for 24 hours were analyzed for lipid peroxidation. Shown are histograms (left) and quantitation (mean±SD of three determinations) (right) of the PE/FITC ratios. **C.** H660 cells left untreated or treated with 2 μM GO-203 for 24 hours were analyzed for GSR transcripts (left). The results (mean±SD of 4 determinations) are expressed as relative GSR mRNA levels compared to that obtained in untreated cells (assigned a value of 1). Lysates were immunoblotted with antibodies against the indicated proteins (right). **D.** Lysates from H660 cells left untreated or treated with 2 μM GO-203 for 24 hours were analyzed for GSH levels. The results (mean±SD of 3 determinations) are expressed as relative GSH levels compared to that obtained in untreated cells (assigned a value of 1). **E.** H660 cells

left untreated or treated with 2 μM GO-203 for 24 hours were analyzed for LRP8 transcripts (left). The results (mean±SD of 4 determinations) are expressed as relative LRP8 mRNA levels compared to that obtained in untreated cells (assigned a value of 1). Lysates were immunoblotted with antibodies against the indicated proteins (right). **F.** Lysates from H660 cells left untreated or treated with 2 μM GO-203 for 24 hours were analyzed for GPX activity. The results (mean±SD of 3 determinations) are expressed as relative GPX4 activity compared to that obtained in untreated cells (assigned a value of 1).

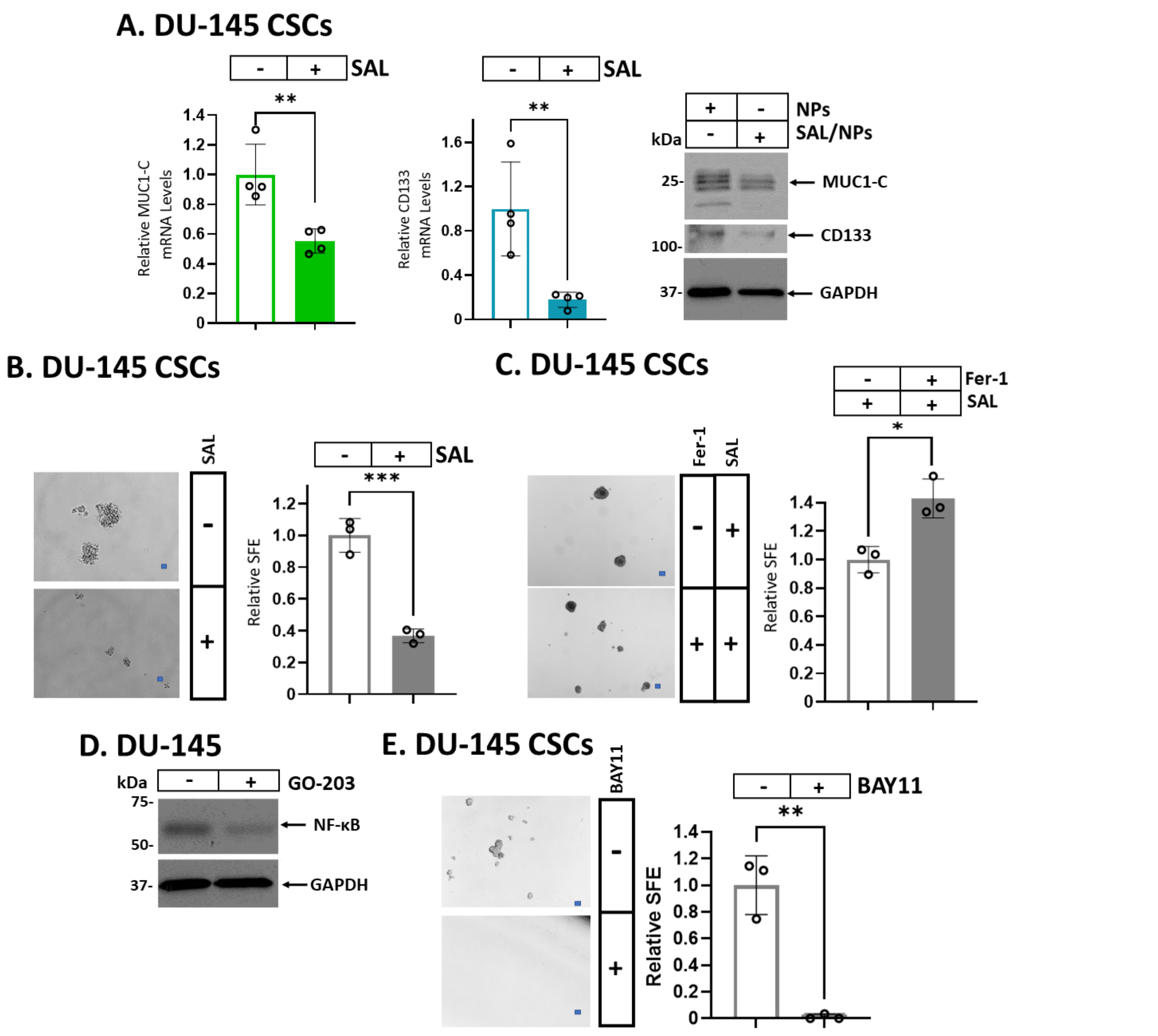

Supplementary Figure S6. Effects of SAL and targeting MUC1-C in CSCs. A. Enriched S8 CSCs treated with vehicle or 1 μM SAL for 24 hours were analyzed for the indicated mRNA levels by qRT-PCR. The results (mean±SD of 4 determinations) are expressed as relative mRNA levels compared to that obtained in vehicle-treated cells (assigned a value of 1)(left). Lysates were immunoblotted with antibodies against the indicated proteins (right). **B.** DU-145 CSCs treated with vehicle or 1 μM SAL for 24 hours were analyzed for tumorsphere formation. Photomicrographs are shown for the treated tumorspheres (left). The results (mean±SD of three determinations) are expressed as relative SFE compared to that obtained in control cells (assigned a value of 1)(right). **C.** DU-145 CSCs treated with 1 μM SAL in the absence and presence of 10 μM Fer-1 for 7 days were analyzed for tumorsphere formation. Photomicrographs are shown for the treated tumorspheres (left). The results (mean±SD of three determinations) are expressed as relative SFE compared to that obtained in SAL alone treated cells (assigned a value of 1)(right). **D.** Lysates from DU-145 cells left untreated or treated with 2 μM GO-203 for 24 hours were immunoblotted with antibodies against the indicated proteins. **E.** DU-145 CSCs treated

with 10 μM BAY11-7082 for 7 days were analyzed for tumorsphere formation. Photomicrographs are shown for the treated tumorspheres (left). The results (mean±SD of three determinations) are expressed as relative SFE compared to that obtained in BAY11-7082 treated cells (assigned a value of 1)(right).

A. DU-145

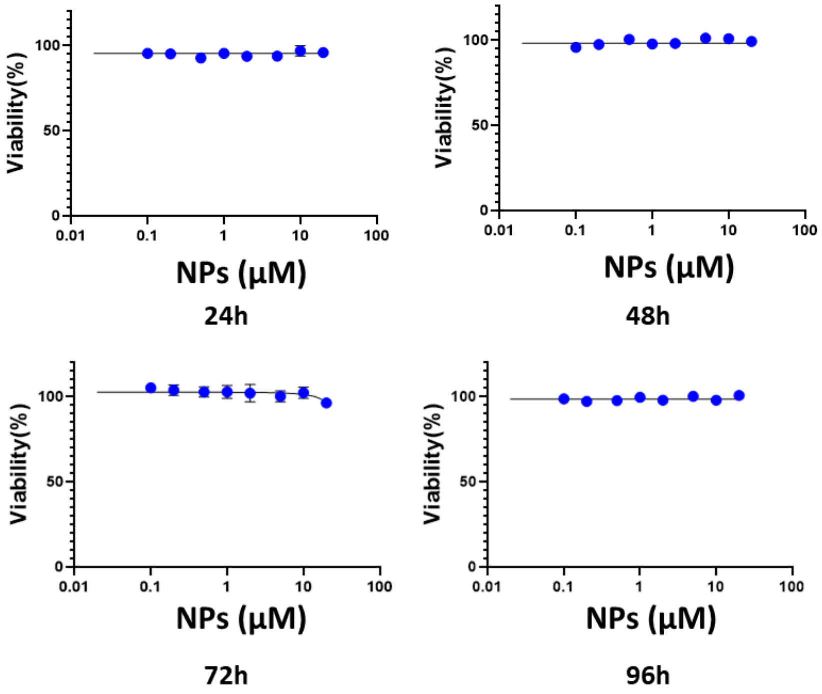

B. DU-145

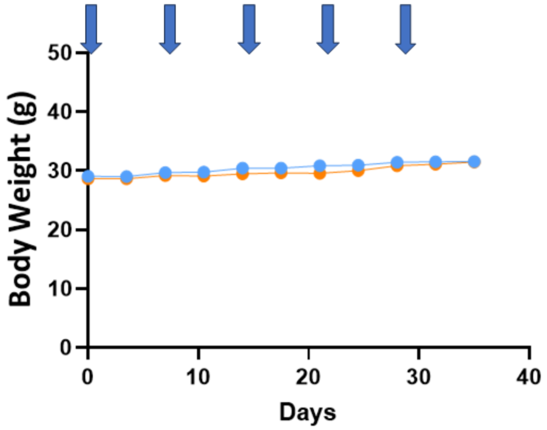

Supplementary Figure S7. Effects of empty NPs. A. DU-145 cells were treated with empty NPs for 24-96 hours at equivalent amounts used in studies of SAL/NPs shown in Fig. 7A. Viability was assessed by Alamar Blue staining. The results (mean±SD of 4 determinations) are expressed as relative viability compared to untreated cells (assigned a value of 100%). **B.** Six-week old nude mice were injected subcutaneously in the flank with 1 × 10^7 DU-145 cells. Mice pair-matched into two groups of 6 mice each when tumors reached 150-200 mm^3 were treated with SAL/NPs or empty NPs each week x 5 weeks. Body weights are expressed as the mean±SEM for six mice.

Supplementary Table S1. Primers used for qRT-PCR.

**MUC1-C**	Forward	AGACGTCAGCGTGAGTGATG
	Reverse	GCCAAGGCAATGAGATAGA
**GSR**	Forward	GGCTTTCCAAGTTGTGAGGG
	Reverse	TATTCCTAAGCTGGCACCGG
**LRP8**	Forward	CCTGCGAGGGTTCATGTATT
	Reverse	GGCTCAGGAAGTCAGTGGAG
**GPX4**	Forward	AGAGATCAAAGAGTTCGCCG
	Reverse	TTGTCGATGAGGAACTGTGG
**CD133**	Forward	AGTCGGAAACTGGCAGATAGC
	Reverse	GGTAGTGTTGTACTGGGCCAAT
**β-actin**	Forward	ACAGAGCCTCGCCTTTG
	Reverse	CCTTGCACATGCCGGAG
**GAPDH**	Forward	CCATGGAGAAGGCTGGGG
	Reverse	CAAAGTTGTCATGGATGACC

Supplementary Table S2. Primers used for qPCR.

**GSR PLS**	Forward	GCAAGGCTCAGTGGTATCTAC
	Reverse	CACTGTAGCCTCAAACTCCTG
**LRP8 PLS**	Forward	GCTGCGTGGAACTTTGAAAC
	Reverse	AAAGGTCTAGCTTCCCGAAAC
