# Supplementary material for: MUC1-C is a target of salinomycin in inducing ferroptosis of cancer stem cells
Source: Cell Death Discov. 2024 Jan 5;10:9. doi: 10.1038/s41420-023-01772-9 (PMC10770371; doi:10.1038/s41420-023-01772-9)

Fig.1D

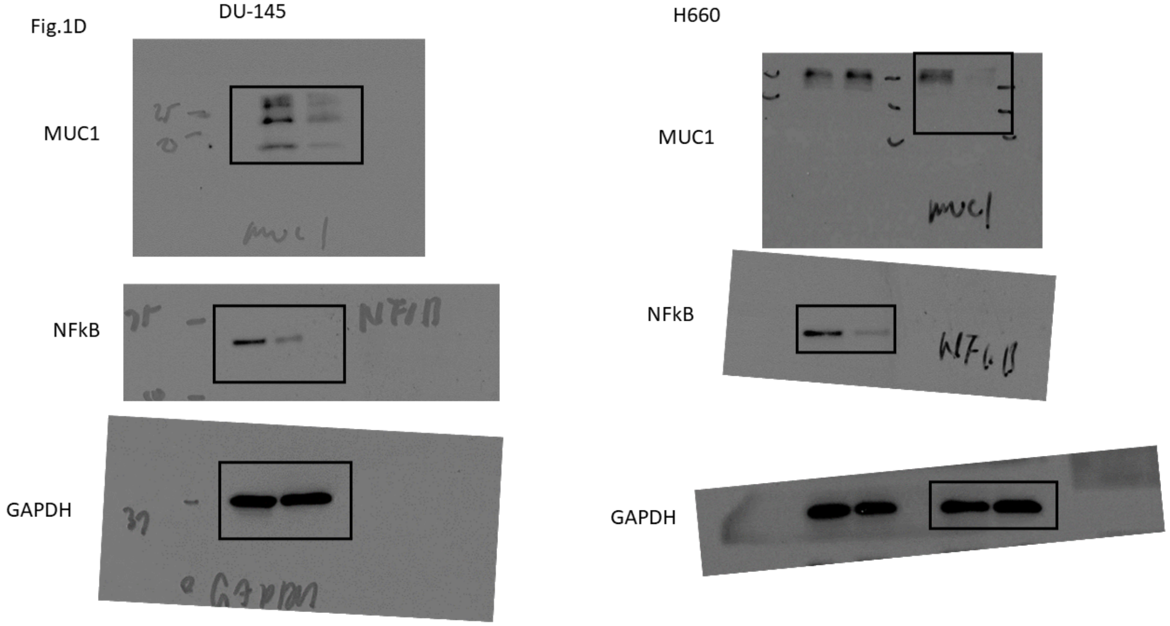

Fig.1E

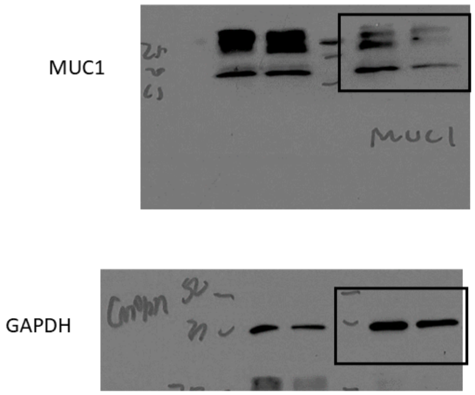

Fig.1F

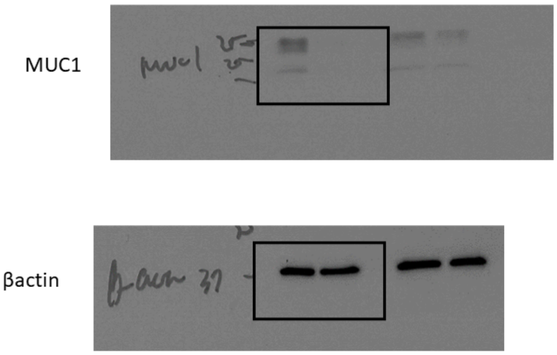

Fig.1G

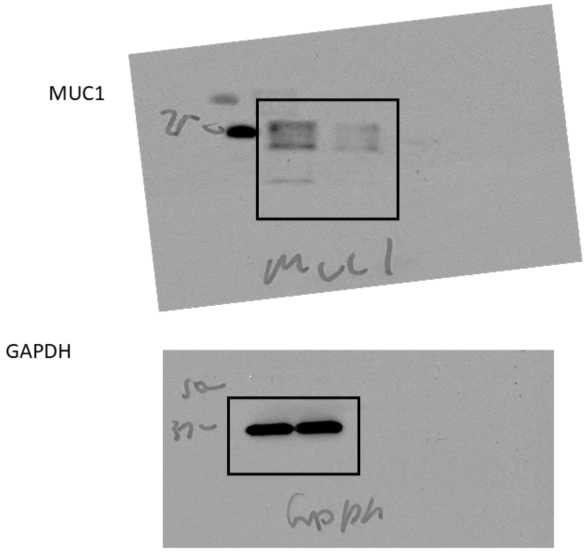

Fig.1H

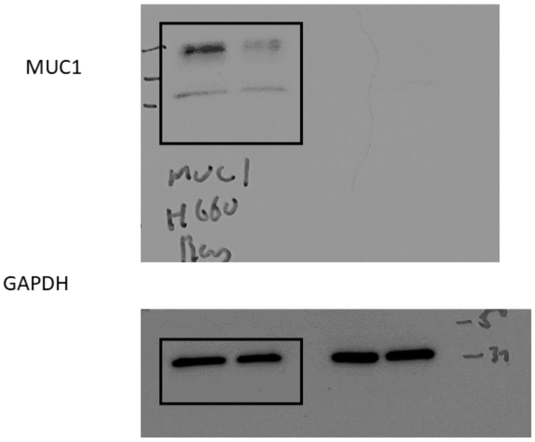

Fig.S1A

MUC1

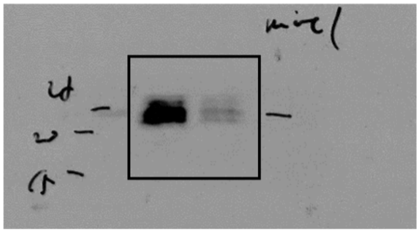

GAPDH

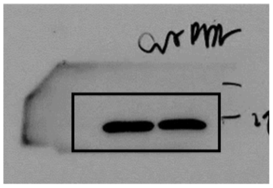

Fig.S1B

MUC1

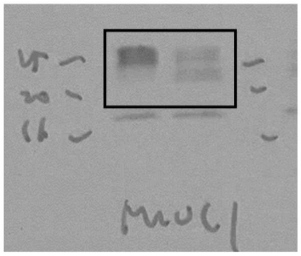

GAPDH

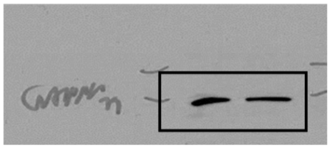

Fig.2F

MUC1

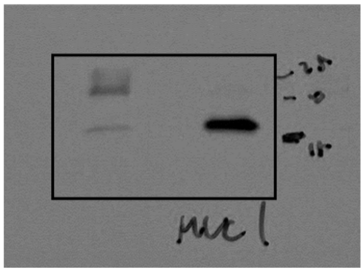

βactin

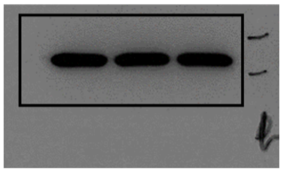

Fig.S2A

MUC1

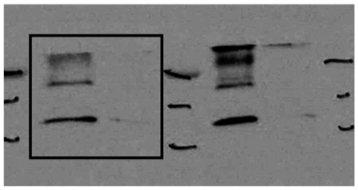

βactin

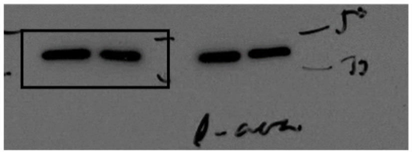

Fig.S2B

MUC1

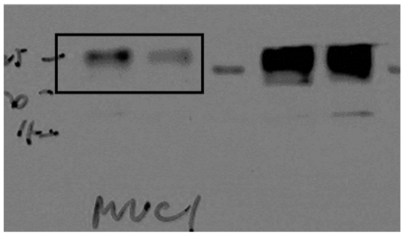

βactin

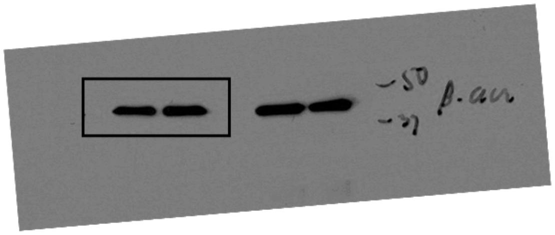

Fig.3A

GSR

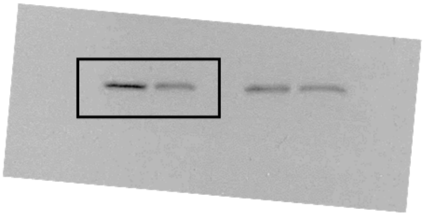

GAPDH

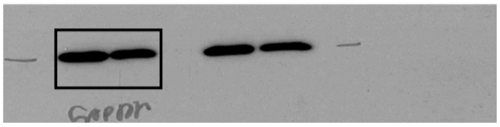

Fig.3B GSR

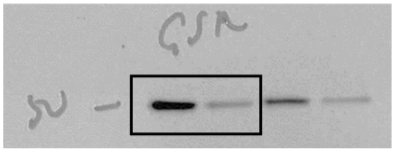

βactin

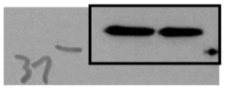

Fig.3E

GSR

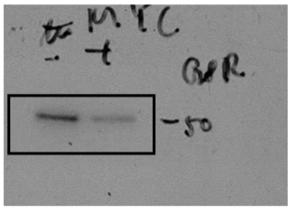

βactin

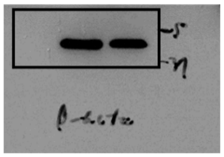

Fig.3G GSR

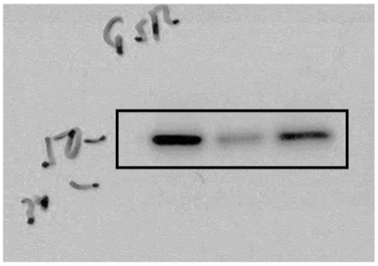

βactin

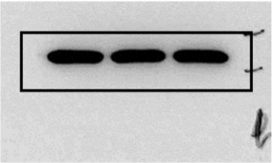

Fig.S3A

GSR

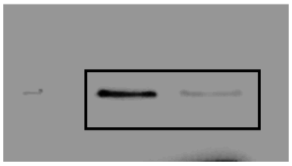

βactin

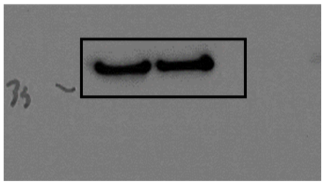

Fig.S3B GSR

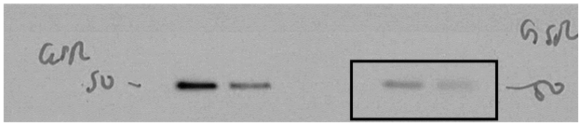

βactin

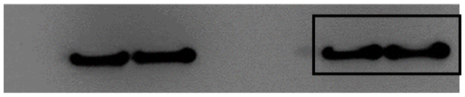

MUC1

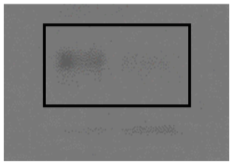

Fig.4A

LRP8

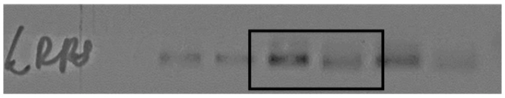

βactin

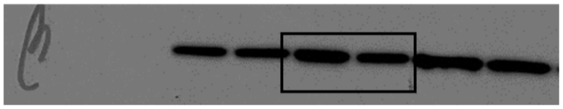

Fig.4B

LRP8

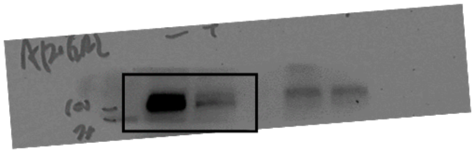

βactin

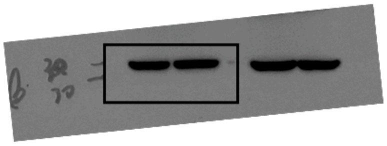

Fig.4E

LRP8

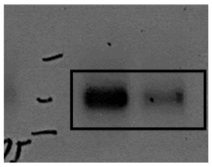

MYC

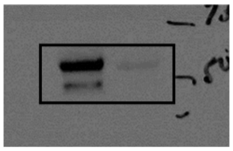

βactin

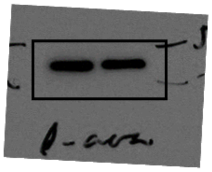

Fig.4F,H

LRP8

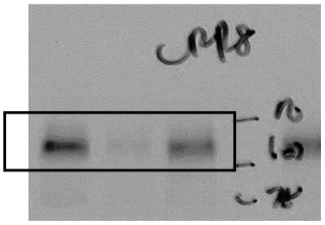

GPX4

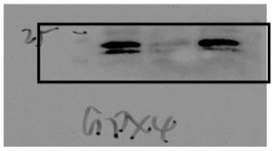

βactin

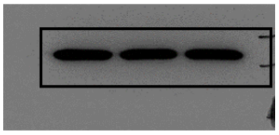

Fig.S4A

LRP8

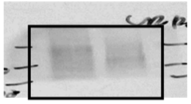

GAPDH

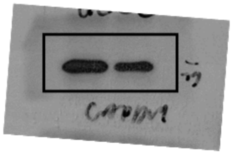

Fig.S4B

LRP8

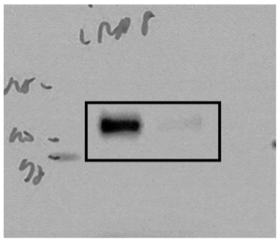

βactin

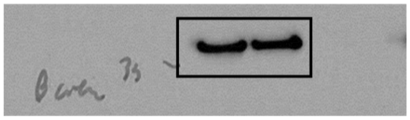

Fig.S6A

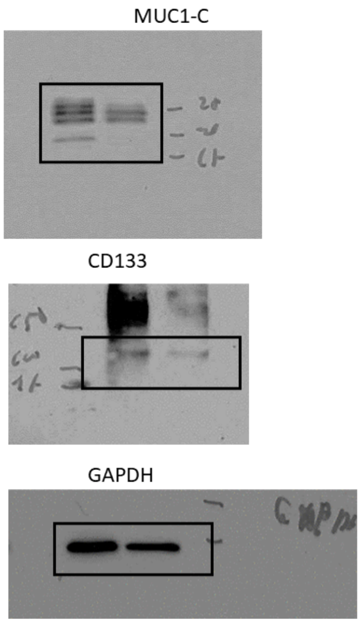

Fig.S6D

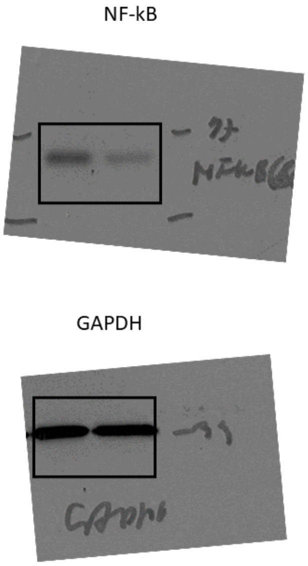

Fig.7B

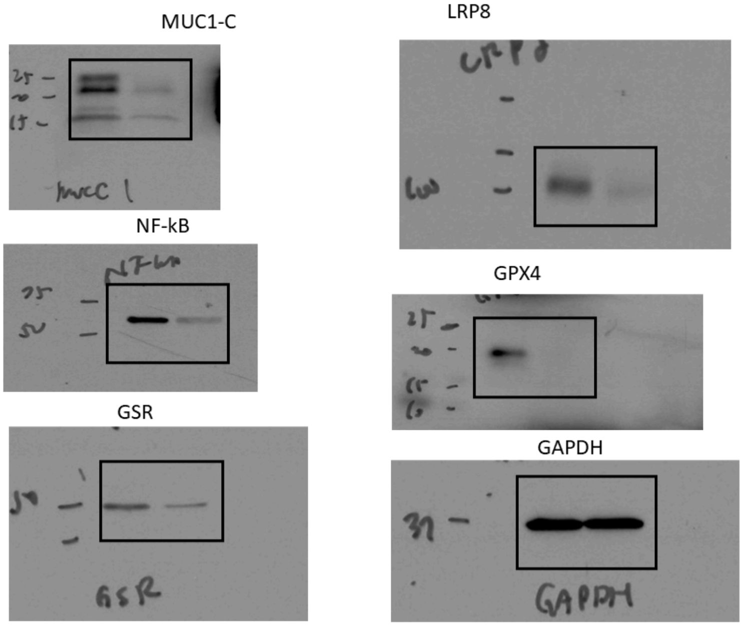

Fig.7E

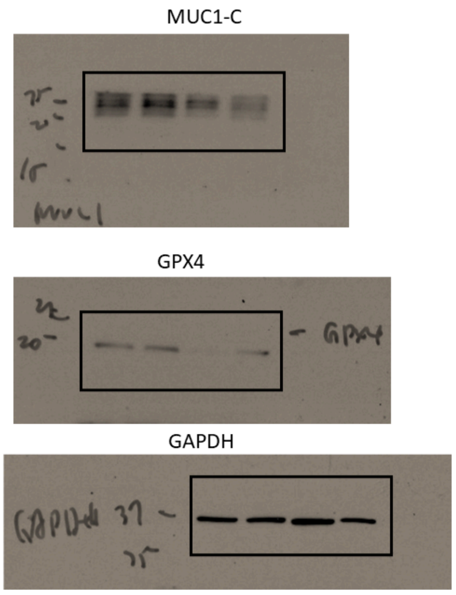

Fig.S4C

LRP8

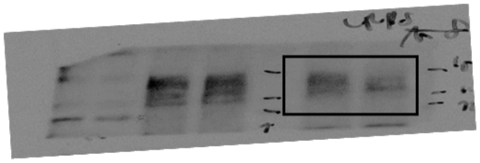

βactin

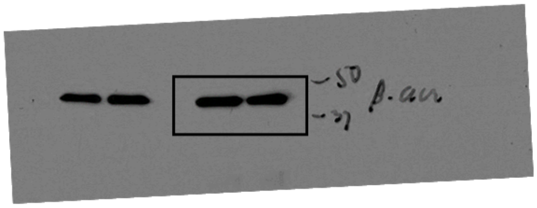

Fig.S4E

GPX4

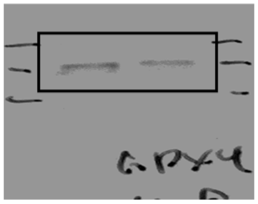

βactin

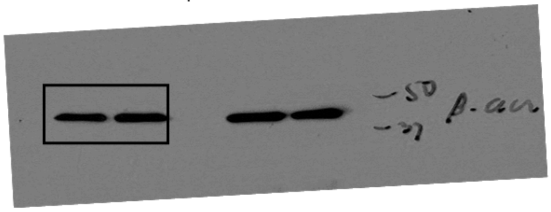

Fig.5D

GSR

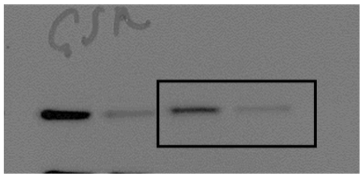

GAPDH

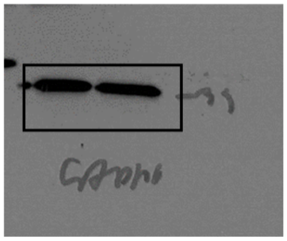

Fig.5F

LRP8

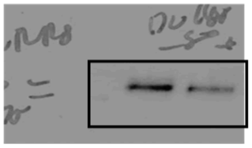

GPX4

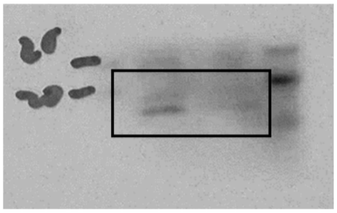

GAPDH

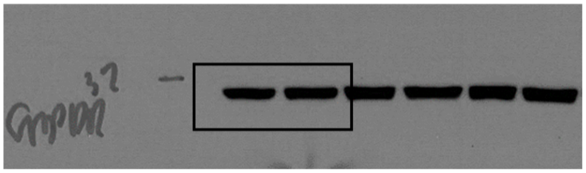

Fig.S5C

GSR

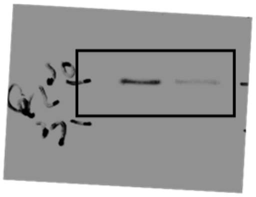

GAPDH

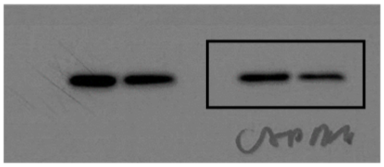

Fig.S5E

LRP8

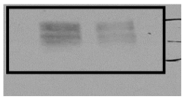

GPX4

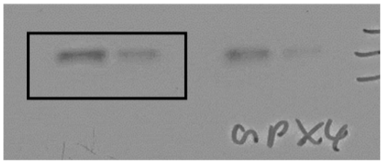

GAPDH

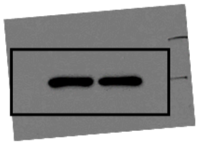

Supplement: Supplementary file 2 — Original Data File [file 41420_2023_1772_MOESM2_ESM.pdf]
